# Prevalence of Risk Factors for Cardiovascular Diseases in Bangladesh: A Systematic Review and Meta-Analysis

**DOI:** 10.1371/journal.pone.0160180

**Published:** 2016-08-05

**Authors:** Kaniz Fatema, Nicholas Arnold Zwar, Abul Hasnat Milton, Liaquat Ali, Bayzidur Rahman

**Affiliations:** 1 The School of Public Health and Community Medicine, Faculty of Medicine, The University of New South Wales, Sydney, Australia; 2 Department of Epidemiology, Bangladesh University of Health Sciences (BUHS), 125/1 Darus Salam, Mirpur, Dhaka-1216, Bangladesh; 3 Centre for Clinical Epidemiology and Biostatistics (CCEB), The School of Medicine and Public Health, Faculty of Health and Medicine, The University of Newcastle, Newcastle, NSW 2008, Australia; 4 Department of Biochemistry and Cell Biology, BUHS, 125/1 Darus Salam, Mirpur, Dhaka-1216, Bangladesh; Azienda Ospedaliera Universitaria di Perugia, ITALY

## Abstract

**Background:**

Given the rising incidence of cardiovascular diseases (CVDs) in Bangladesh, an improved understanding of the epidemiology of CVD risk factors is needed. Therefore, we reviewed published studies on CVD modifiable risk factors e.g., Type 2 Diabetes Mellitus (T2DM), hypertension (HTN), dyslipidemia and smoking as well as studies on CVDs and conducted a meta-analysis of risk factors and disease prevalence.

**Methods:**

We searched the GLOBAL HEALTH, MEDLINE, EMBASE ‘BanglaJol’ databases for all studies in English on CVDs and its associated modifiable risk factors. Random effects meta-analysis methods were used to estimate pooled prevalence.

**Results:**

There were 74 eligible studies (outcome: T2DM = 32, HTN = 24, dyslipidaemia = 8 and smoking = 25; CVDs = 10). Due to high between study heterogeneity (p<0.001, I^2^> 95%) in the prevalence of CVD risk factors, we presented median and interquartile range (IQR) instead of the pooled estimates as the summary measures. Median (IQR) prevalence of T2DM, HTN, dyslipidemia and smoking were 5.9% (1.97%-8.25%); 15.1% (10.52%-17.60%); 34.35% (10.66%-48.50%) and 40.56% (0.80%-55.95%), respectively. The prevalence of T2DM and dyslipidemia were significantly higher in urban compared to rural populations (13.5 vs 6%, p<0.001; 41.5 vs 30%, p = 0.007, respectively).

**Conclusions:**

The prevalence of risk factors for CVDs is high in Bangladesh, more so in urban areas. Ageing of the population may be a factor but urbanization seems to have an influence, possibly related to changes in dietary and physical activity patterns. Further research, in particular longitudinal studies, is needed to explore the complex interaction of these factors and to inform policies and programs for the prevention and management of CVDs in Bangladesh.

## Background

The burden of cardiovascular diseases (CVDs) is rising in developing countries, particularly low and middle income countries (LMICs), creating a major challenge for the health sector. According to the World Health Organization (WHO) CVDs were the cause of 17.5 million deaths (31% of all death) around the world in 2012, of which 80% occurred in LMICs [1], and 85% of all global disability arose from CVDs [2].

CVDs and its associated known risk factors account for 13.4% of disability adjusted life years (DALYs) lost in Bangladesh [3]. The major CVD risk factors such as abnormal glucose metabolism, high blood pressure, dyslipidemia, smoking, along with increasing age are well established [2, 4]. Obesity constitutes major risks for CVDs both directly (through underlying insulin resistance and inflammatory changes) and indirectly (through the effect on other immediate risk factors like T2DM, dyslipidemia and HTN). After China and India, Bangladesh has the highest prevalence of diabetes mellitus (DM) among LMICs (8.4 million or 10% of the population) and the prevalence could increase by 13% by 2030 [5]. According to the INTERHEART study Bangladeshis had the highest prevalence of CVD risk factors among five South Asian countries with the prevalence of self-reported history of hypertension (14.3%), abdominal obesity (43.3%), current and former smoking (59.9%), and the lowest prevalence for regular physical activity (1.3%) and daily intake of fruits and vegetables (8.6%) [6]. In Bangladesh, 99.6% male and 97.9% females are exposed to at least one of the established risks of CVDs and at risk of CVD at a younger age (below 40 years in men) [3, 7, 8].

Considering the increasing trend of CVD related mortality in Bangladesh there is a need to understand the epidemiology of CVDs and its risk factors in the country. A few review studies were conducted on the effect of type 2 diabetes mellitus (T2DM) and hypertension (HTN), but not on other risk factors of CVD [9–11]. This systematic review explores the prevalence of major CVD risk factors (T2DM, HTN, dyslipidemia and smoking) and their correlates in Bangladesh with the aim of describing the overall epidemiology of CVD risk factors in Bangladesh.

## Methods

### Search strategy

Systematic literature search initially was conducted on the EMBASE, GLOBAL HEALTH, MEDLINE databases and a country specific search engine “BanglaJol”. The search was limited upto August 2014, without any language restrictions. In Embase, we inputted location: South Asia and Bangladesh; and for outcome: “cardiovascular disease, diabetes mellitus, hypertension, dyslipidemia and smoking” and explored all fields. The “OR” Boolean operator was used to combine within the outcome and used “AND” to combine across outcome and location. After that instead of South Asia AND (“Bangladesh”[Emtree Terms] Explored [All Fields]). In addition we also searched for obesity using OR (“obesity”[Emtree Terms] Explored [All Fields]). We also conducted a hand search to find articles referenced in other studies.

### Data extraction

We extracted the following information for each reviewed study and recorded data (summary included in S1–S6 Tables) as follows:

1. *General information*: First author’s name and publication year, title and journal name, study place and location (i.e., urban or rural) and year of data collection,
2. *Study design*: Type of study, sampling pattern (i.e., random or non-random), sample size and sample characteristics such as age, gender and race,
3. *Study factors*: prevalence of T2DM—fasting plasma glucose (FPG), and impaired glucose tolerance (IGT); HTN–systolic and diastolic blood pressure (sHTN and dHTN); dyslipidemia–lower level of high density lipoprotein (HDL), and high level of total cholesterol, and high level of total triglyceride (TG); Obesity—body mass index (BMI), waist circumference (WC), waist hip ratio (WHR); and waist height ratio (WHtR) and smoking status with diagnostic criteria,
4. *Analysis*: calculation of prevalence with confidence intervals (CI) and p values.

### Quality assessment

There is no standard quality assessment instrument for assessing the quality of cross-sectional studies reporting prevalence of a condition. Based on the quality assessment criteria for observational epidemiological studies [12] we developed a checklist to categorically assess the qualities of our included studies. The study quality was assessed in three domains: 1) definition of study base 2) representativeness of the sample and 3) accuracy of outcome measurements. In each domain the studies were categorised as of good, moderate or poor quality. The study base was considered good if it was defined appropriately in terms of person, place and time of the study population. If any of these criteria (i.e., person, place and time of study population) was not clearly stated, the study base was considered moderately defined and if none of these was clearly stated the study was deemed to have poor base.

If the study participants were selected randomly form the general population (not from a hospital) with defined age group, the sampling was considered good. If the samples were selected purposively from the general population the samples was thought to be of moderate quality. Non-random hospital based samples were considered of poor quality.

Studies with good outcome measurement clearly stated the methods and used evidence based established technique for the measurement. For example, a good quality study would diagnose diabetes mellitus by using classification system of WHO or the American Diabetes Association (ADA) or International Diabetes Federation (IDF) based criteria. Whether the test was based on fasting [13] or 2-hr post-prandial glucose [14] or the specimen was either whole blood or plasma. Studies with self-reported diabetes were considered of poor quality.

We did not estimate overall study quality because its use is controversial [15]. We used the domain specific quality information to conduct sub-group meta-analysis in order to examine heterogeneity.

### Statistical analysis

We used ‘METAPROP’ command in Stata version 12.1 (StataCorp LP, College Station, TX) to perform fixed and random effects meta-analysis of proportions. This routine provides procedures for pooling proportions in a meta-analysis of multiple studies and/or displays the results in a Forest plot. The confidence intervals are based on score (Wilson)[16] or exact binomial (Clopper-Pearson) [17] procedures. Along with the pooled estimate this command also reports the significance test of it and the between study heterogeneity. The heterogeneity is also quantified using the I^2^ statistics [18]. We considered low, moderate and high degrees of heterogeneity based on the values of I^2^ as <25%, 25–75% and above 75% respectively [19]. Sub-group analyses were performed according to criteria for disease diagnosis (e.g., for DM using both fasting and 2hBG (2 hours blood glucose), fasting BG (blood glucose) only and only after glucose (AG). We also compared pooled estimates across sub-groups using chi-squared test.

## Results

### Study selection

**Fig 1:**
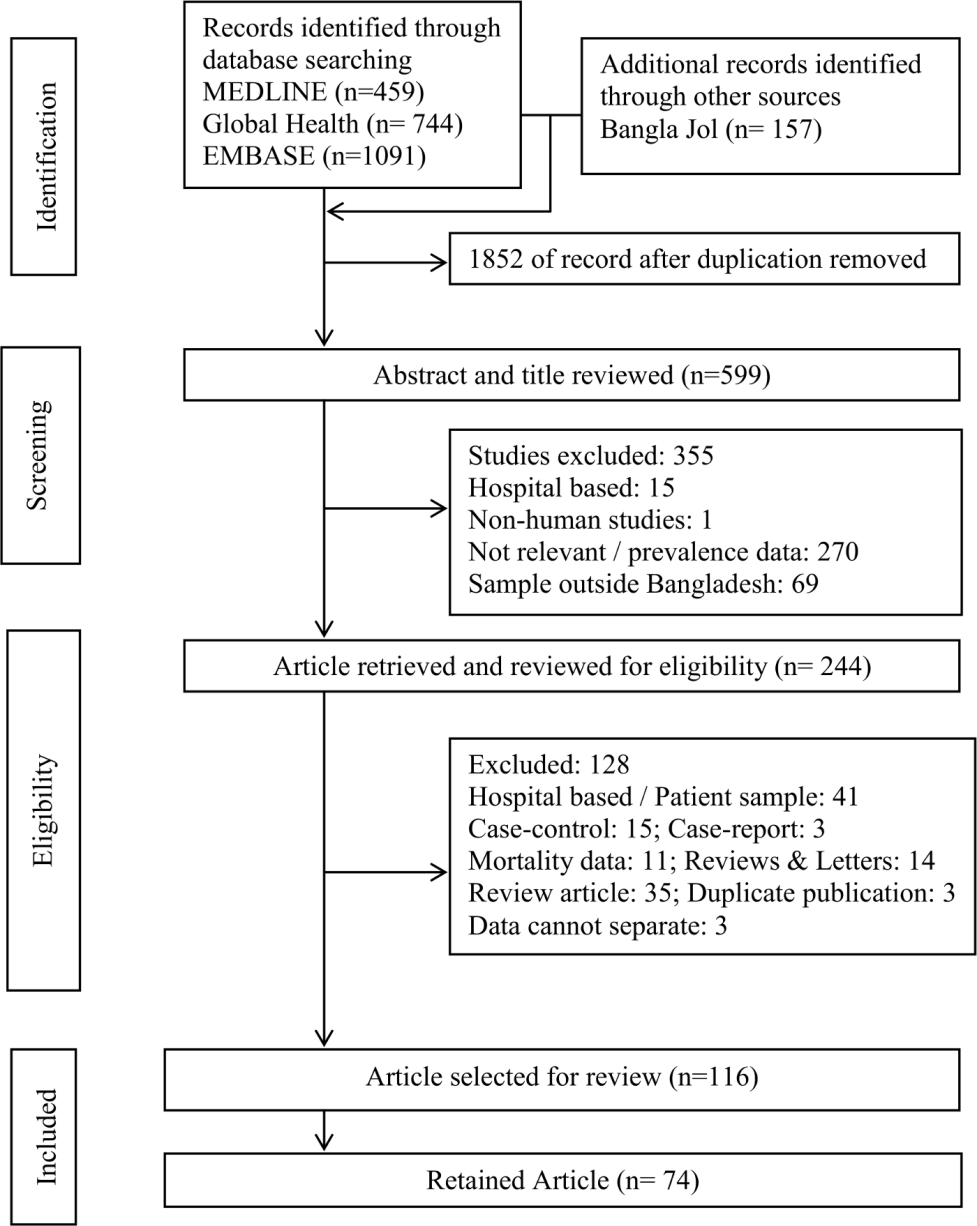
PRISMA diagram of the literature screening process. Numbers indicate the article count retained at each step of the process.

For all the articles that met the identification criteria, primary investigator (KF) initially screened the titles and abstracts. The inclusion criteria for this screening were: i) prevalence or incidence data available on either CVD or T2DM or HTN or smoking or dyslipidemia, ii) published result up to August 2014. Initially after removal of duplication, 599 articles were identified as potentially eligible. Among these, 355 studies were excluded for the following reasons: based on hospital data (n = 15) as the source population could not be identified to estimate prevalence; not relevant with our objectives or had no prevalence data (n = 270), sample not from Bangladeshis or Bangladeshi population living overseas (n = 69); and one study from a non-human sample. Following review of the full text a total of 128 studies were excluded for the following reasons: 41 were collected from hospital admitted patients or hospital registry or samples of patient with particular diseases; articles based on study designs that did not allow to estimate prevalence, eg., case-control (n = 15); case report (n = 3); mortality (n = 11); review article (n = 35); review and letters to editor (n = 14); duplicate publication (n = 3); and data cannot be separated from the table (n = 3). The remaining 116 articles were further examined. The set criterion for inclusion in the meta-analysis was that the participants should be selected from a general population and not from a hospital populations. Therefore, a total of 74 studies (S1–S6 Tables) were used to calculate the pooled prevalence. Studies conducted on special populations (e.g., elderly, pregnant), being hospital/ clinic-based, studies reporting the results of larger studies as duplications were excluded from the meta-analysis (Fig 1).

### Overall characteristics of the reviewed studies (n = 74)

Nationally representative data are coded from recent studies that come from the Bangladesh Demographic and Health Survey (DHS) carried out in 2011. However the majority of these studies were mostly conducted in Dhaka, followed by a limited number of studies in Chittagong, Sylhet and Rajshahi. Therefore the data is limited to these four divisions out of seven divisions in Bangladesh (S1 Fig).

Seventy percent of studies (n = 53) selected participants with a random procedure, implemented at one of three levels: village/city ward, household, or individual. Another 12 studies enrolled everyone who resided in a chosen geographic area and met the study eligibility criteria. Six studies used a non-random sample and information on the sampling strategy could not be ascertained for the remaining three studies. More than 80% of the studies (n = 61) selected the sample from the general population. However, sampling from various types of working groups as well as from tribal populations was also reported. The lower age limit for eligibility was set to 20 years in most of these studies, except in three studies where it was 10 years, 15 years and 18 years, respectively. The sample of the remaining ten studies came from older adults (age>50, n = 3), working professionals (n = 5), and tribal population (n = 2). Sixty one studies had both male and female participants, 2 had only women, 8 had only men and three did not provide information on gender. Forty (52%) studies were conducted in rural areas, 20 in urban areas (27%), and 16 had both urban and rural samples (21%). However considering the present epidemiological transition pattern in Bangladesh, the rural populations mentioned in these studies were recruited from areas closer to urbanised areas and they can be more accurately described as urbanizing rural.

### Operational definitions and overall outcome of the reviewed studies

The World Health Organization, WHO-1980, the WHO -1999, or the American Diabetes Association, ADA -1997 classification system were used to define T2DM for all studies [20, 21]. Its definition varied according to whether the test was either a fasting or 2-hr postprandial glucose, or whether the specimen was either whole blood or plasma. T2DM was self-reported in three studies and not defined in another study. Two studies had gestational diabetes mellitus (GDM) as its outcome.

HTN was also defined in various ways across studies: (i) systolic blood pressure (SBP) ≥140 mmHg or diastolic blood pressure (DBP) ≥90 mmHg (n = 6), (ii) SBP ≥140 and/or DBP ≥90 and/or anti-hypertensive medication use (n = 4), (iii) SBP ≥140 and DBP ≥90 (n = 1), (iv) SBP ≥140 (n = 1), (v) DBP ≥90 or medication use (n = 2), (vi) SBP ≥135 or DBP ≥85 (n = 2), (vii) self-reported (n = 2), (viii) not specified (n = 4) (ix) by clinical staff (n = 1).

The definition of dyslipidemia varied across studies: (i) TG ≥ 1.70, HDL <1.04 for men and <1.29 for women (n = 1), (ii) TG ≥ 1.7, HDL <1.03 for men and <1.29 for women (n = 1), (iii) high cholesterol>240mg/dl, high LDL>160 mg/dl, Low HDL <40mg/dl, high TG ≥200 mg/dl and total Chol/total HDLC (>5.5) (n = 1), (iv) High cholesterol >200mg/dl, Low HDL <40mg/dl, High TG ≥150 mg/dl (n = 1), (v) self-reported (n = 1), (vi) not specified (n = 3).

The definition of current smoking was also confirmed through further questions including information of past use, duration of use, age at start, number of sticks per day.

Only T2DM was reported in 13 studies, HTN as a sole outcome in 4 studies, only dyslipidemia in three studies and obesity two studies. Both T2DM and HTN were in three studies, obesity and lipid abnormalities along with T2DM and HTN were in 11 studies. HTN or T2DM with CVD were in two studies and chronic kidney disease or arsenic with six studies. Cardiovascular disease was considered as solitary outcome for six studies. Four studies coded self-reported data only.

### Estimates of prevalence

All the estimates are presented in the Forest plots. In these plots, each rectangle in the centre represents the point estimates for individual study; the rectangle’s size corresponds to the point estimates weight in the pooled estimate and the horizontal line through the rectangle represents width of the 95% confidence interval (CI) about the point estimate. Each diamond represents a pooled estimate; its vertical axis marks the point estimated and its horizontal extremes the upper and lower 95% confidence bounds. Studies that had undue influence on the pooled estimate were excluded from the forest plots [22]. We got very high between study heterogeneity (p<0.001, I^2^> 95%) was reported in the prevalence of CVD risk factors. Therefore, we preferred median and interquartile range (IQR) to the pooled estimates as the summary measures.

#### T2DM (n = 32)

**Fig 2:**
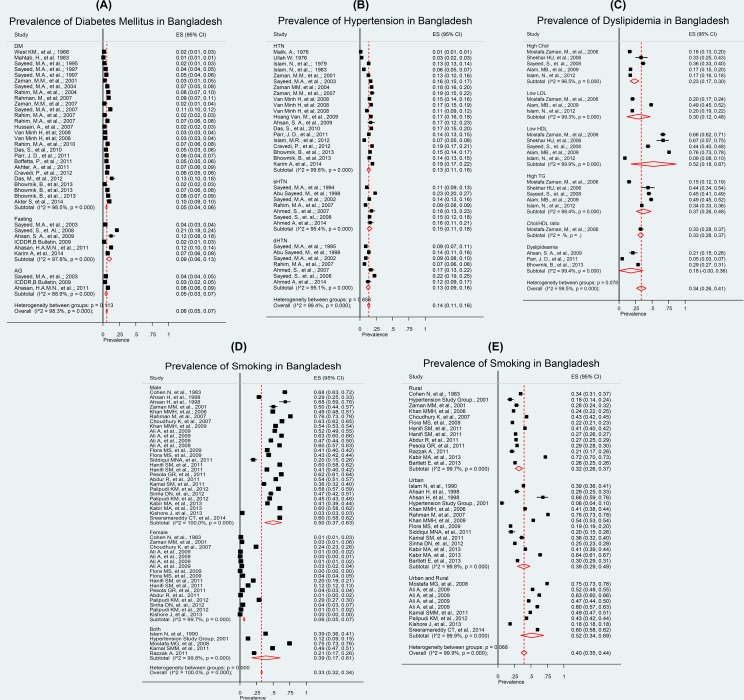
**a: Prevalence of diabetes mellitus in Bangladesh; b: Prevalence of hypertension in Bangladesh; c: Prevalence of dyslipidemia in Bangladesh; d: Prevalence of smoking in Bangladesh (according to gender); e: Prevalence of smoking in Bangladesh (according to study places)**.

Overall median prevalence was 5.9%, IQR: 1.97%-8.25% and pooled prevalence of 7.0%, (I^2^ = 99.0%, p<0.001). The very high heterogeneity relates to the findings from two studies. One of these reported prevalence in three geographic areas [23], one of which was very high. The other study [24] used a stratified sampling method (e.g., participants through a multi-stage random sampling procedure, from a middle-income neighbourhood in the capital city of Dhaka). If we exclude these two study findings & re-analyse, the pooled prevalence within strata based on DM (diagnosed from both F and 2hBG) becomes 5.0% (95% CI: 4.0%–6.0%, I^2^: 98.3%). Within strata based on fasting the pooled prevalence for T2DM was 9.0% (95% CI: 6.0%–13.0%, I^2^: 97.8%) and based on after glucose tolerance test it was 5.0% (95% CI: 3.0%–7.0%, I^2^: 88.9%). Overall T2DM prevalence estimate was 6.0% (95% CI: 5.0%–7.0%, I^2^: 98.0%) (Fig 2A).

#### HTN (n = 24)

Overall median prevalence was 15.1%, IQR: 10.52%-17.60% and pooled prevalence of both systolic HTN (sHTN) and diastolic HTN (dHTN) was 13.0% (95% CI: 11.0%–16.0%) with I^2^: 99.6% and p<0.001. Based on systolic blood pressure only, the pooled prevalence was 15.0% (95% CI: 11.0% to 18.0%, I^2^: 69.1%) and based on diastolic blood pressure, it was 13.0% (95% CI: 9.0%–16.0%, I^2^: 95.8%). Overall the HTN prevalence estimates was 14.0% (95% CI: 11.0%–16.0%, I^2^: 99.5%) (Fig 2B).

#### Dyslipidemia (n = 8)

Overall median prevalence was 34.35%, IQR: 10.66%-48.50% and pooled prevalence for high cholesterol was 23.0% (95% CI: 17.0%–30.0%) with I^2^: 96.5% and p<0.001). The prevalence of elevated LDL from one study in rural areas was 30.0% (95% CI: 12.0%–44.0%). However the low HDL pooled prevalence was 52.0%, with I^2^ = 99.9% and p<0.001. As the defined cut off value for low HDL and laboratory procedure varied we kept the result as it is. Prevalence of High TG and cholesterol/HDL ratios (from one study) were 37.0% (95% CI: 26.0%–48.0%, I^2^: 98.4%) and 33.0% (95%CI: 28.0%–37.0%). Overall dyslipidemia pooled prevalence was 18.0% (95% CI: -0.0%– 31.0%, I^2^: 99.4%). High heterogeneity was noted and we observed three studies reported very high prevalence of low HDL which stands out from of all other studies and the study covered both urban and rural population [25–27]. Overall dyslipidemia prevalence estimate was 34.0% (95% CI: 24.0%–43.0%, I^2^: 98.9%).

In addition, we also observed obesity although it is not an established risk factor for CVD as are the others.

#### Obesity (n = 15)

Considering BMI as obesity indicator, median prevalence (cut-off value >25.01) was 16.02%, IQR: 7.9%-47.3% and pooled prevalence was 15.0% (95% CI: 11.0%–20.0%) with I^2^: 99.7% and p<0.001. This is due to a study with very high prevalence that included both urban and rural and the pooled prevalence became 10.0% (95% CI: 8.0%–12.0%, I^2^: 98.7%). Pooled prevalence of obesity defined by WC (>0.90 male, >0.80 female) was 21.0% (95% CI: 13.0%–28.0%). Obesity defined by WHR (>1.0 male, >0.85 female), a study [28] reported very high prevalence among rural participants and after excluding it, pooled prevalence was 55.0% (95% CI: 47.0%–62.0%, I^2^: 99.3%). Prevalence of waist to height ratio (>0.5) from one study was 60.0% (95% CI: 58.0%–62.0%). Considering obesity from any of the markers, we estimated overall obesity, the pooled prevalence was 24.0% (95% CI: 18.0%– 30.0%, I^2^: 99.9%) (S2 Fig).

#### Smoking (n = 25)

Overall median prevalence of smoking was 40.56%, IQR: 0.80%-55.95% and pooled prevalence was 33.0% (95% CI: 32.0%–34.0; I^2^ = 100% and p<0.001). Among males, the pooled prevalence was 50.0% (95% CI: 37.0%–63.0%, I^2^: 100%) and females it was 6.0% (95% CI: 5.0%–7.0%, I^2^: 99.7%). From combined gender studies the prevalence estimates was 39.0% (95% CI: 17.0%–61.0%, I^2^: 99.8%). However pooled prevalence based on area-wise smoking was 40% (95% CI: 35.0%–44.0%). In the rural areas, the pooled prevalence was 35.0% (95% CI: 28.0%–41.0%, I^2^: 99.6%) and in the urban areas, it was 44.0% (95% CI: 31.0%–57.0%, I^2^: 99.4%). Pooled estimate, from studies reporting prevalence in urban and rural areas combined, was 40.0% (95% CI: 34.0%–47.0%, I^2^: 99.9%).

To explore the cause of heterogeneity, a quality assessment (as per study base, method of sample selection and measurement of outcome) of the studies was performed (Table 1). Initially we conducted a subgroup (i.e., poor, moderate and good) analysis and meta-regression to examine the difference in pooled estimates among study qualities for T2DM, HTN, dyslipidemia and obesity. We did not find a significant effect of these quality domains on the pooled estimates for the prevalence of T2DM, HTN, dyslipidemia and smoking.

**Table 1:** Quality assessment of the studies and area wise distribution of the published papers.

*Phase 1:	**Study base (%)**			**Sample selection Method (%)**			**Outcome measurement (%)**		
	**Good**	**Moderate**	**Poor**	**Good**	**Moderate**	**Poor**	**Good**	**Moderate**	**Poor**
Diabetes (n = 28)	29	43	29	25	36	39	86	3	11
Hypertension (n = 24)	17	58	25	17	46	38	79	13	8
Dyslipidaemia (n = 8)	12.5	37.5	50	-	38	62	62.5	25	12.5
Smoking (n = 25)	17	37.5	46	29	29	42	46	46	8
CVD (n = 10)	30	40	30	-	50	50	70	20	10
^*†*^***Phase 2*:**	**Urban (%)**		**Rural (%)**		**Urban and rural (%)**			**Semi urban (%)**	
Diabetes (n = 28)	25		60.7		10.7			3.6	
Hypertension (n = 24)	25		67		8			-	
Dyslipidemia (n = 8)	50		37.5		12.5			-	
Smoking (n = 25)	24		36		40			-	
CVD (n = 10)	10		50		40			-	

* Study quality evaluated by following the checklist for this study.

^***†***^ Area wise selected study distribution.

We also explored the cause of heterogeneity according to study population (i.e., semi urban, urban, rural, and both) following the same steps as study quality. We observed a significant effect of the study population on the prevalence of T2DM. In the urban areas, the prevalence was three times higher than rural areas (p<0.001) and for dyslipidemia, it was 2.5 times higher (p = 0.007). No significant effect of area was observed on the prevalence of HTN (p = 0.31). For smoking, there was no difference between rural and urban areas but combined urban and rural studies showed more than two times higher prevalence than rural areas (p<0.010). No trend analysis has been performed due to lack of sufficient data for any risk factor. Sensitivity analyses have been done with age group and sex but no difference was found except smoking. Therefore, the results are not presented.

### Assessment of variables associated with CVD risk factors and CVDs

There were 19 studies (excluding two studies for duplicate publication) that reported T2DM- related covariates information. These studies reported the prevalence of diabetes was positively associated with male gender, age, family history of DM, higher income or socioeconomic class, physical activity level / sedentary lifestyle, BMI, WHR, waist girth, WHtR, and as a whole, general and central obesity, SBP, hypertension, total cholesterol and triglyceride.

Twelve studies reported covariates of HTN-related data. HTN showed significant positive association with increased age, female gender, 2h BG / hyperglycaemia, higher educational status, urban areas, upper socio-economic class, BMI, WHR, WHtR, overall obesity, animal protein, dietary pattern (concentrations of salt, sugar, trans-fats and saturated fats in manufactured food products). Longitudinal studies that were conducted in the arsenic contaminated areas showed positive association between HTN and high arsenic concentration in drinking water or duration of exposure with a dose-response relationship. However, none of the articles of dyslipidemia and obesity related to the risk of CVDs reported data on their covariates.

Only 13 studies reported covariates of smoking related data. Smoking showed significant positive association with increased age, lower level of education, unemployment status, place of residence, marital status, having STDs, premarital and extra marital sex, religion [non-Muslim (i.e., Hindu/ Christian/ others)], illegal drug use, poverty.

Covariates data of CVDs have been reported in four studies. Among those, three of the studies reported coronary artery disease and the fourth study included stroke in addition to this. Hypertension, age, central obesity defined by WHR, male gender, social class, 2h after blood glucose, albumin creatinine ratio (ACR) and family history were positively associated with CVDs.

## Discussion

The present study systematically evaluates cross sectional studies on the prevalence of CVD risk factors and the prevalence of CVDs among the Bangladeshi population. The pooled prevalence estimates obtained for T2DM and HTN were similar to a previous study [9]; that is, 5.9% and 15.1%, respectively. For dyslipidemia and smoking it was 34.35% and 40.56%, respectively. Both T2DM (p<0.001) and dyslipidemia (p = 0.007) were higher in urban than rural areas.

T2DM has been better studied in Bangladesh compared to the other immediate CVD risk factors. The pooled prevalence from these studies is significantly lower than the recent nationwide survey in adults (10%) [29]. Multiple factors, such as method of blood sugar measurement for diabetes and diagnostic criteria used for T2DM, are likely to explain this variation. We found that the prevalence of T2DM ranges from 5%–7% in rural areas whereas, 10%–17% in urban areas. The rural prevalence is slightly higher than that in South India and Pakistan (less than 5%), but it is considerably higher than China (3%) [30]. The differences in prevalence’s among various populations become less prominent when those are estimated in urban settings (Sri Lanka 6%–14%, South India ~12%) [30]. Indian migrants in Mauritius, Fiji, Singapore and Tanzania, who adopted a more urbanized life showed a greater prevalence (15%–20%) [30].

The prevalence of HTN was found to be 10%–16% in rural Bangladeshi people, slightly higher (12%–25%) in urban areas and, overall, 13%-18% among the adult population. This is less than South Asian countries such as Bhutan (23.9%), India (31.45%), Nepal (31.5%), Pakistan (33.8%), and Sri Lanka (25%) [12].

In contrast to marked differences in rural-urban distribution of HTN, the pooled prevalence of dyslipidemia (with any one lipid being abnormal) was only slightly different, e.g., 8%–42% in rural and 34%–49% in urban areas. Analysis of individual lipids shows pro-atherogenic profile (high cholesterol and high TG). However, lack of reliable data on LDL and HDL precludes any conclusion on these two lipids, especially on the LDL/HDL ratio which is considered as one of the major determinants of CVD risk. High dyslipidemia markers have been observed among South Asians, including Bangladeshis, when compared with the European and Chinese populations in a Canadian study [31]. Additionally, overweight and obesity constitute major risks for CVDs both directly and indirectly. It is evident that generalized obesity (predominantly measured by BMI) is still low in Bangladesh, in both rural (18%–36%) and urban (18%–28%) areas. However, measurement of central obesity by using waist circumference shows a higher value (13%–28%) and its assessment by WHR shows an alarmingly high value (45%–57%) (S2 Fig). We found the rural and urban pooled prevalence to be almost similar (18%–36% vs 18%–28%).

Tobacco consumption has been relatively well studied among Bangladeshis. While only 5%–7% females (both in rural and urban areas) are smokers, the proportion among males is high (37%–63%). The proportions do not differ markedly between urban and rural areas (29%–49% vs 26%–37%). Smokeless tobacco use (ingested with betel leaf or chewed or sniffed as powder) was common, particularly among the poor and in rural areas, irrespective of gender [32, 33]. Studies on the strength of the association of tobacco consumption, particularly smokeless tobacco, with CVDs, however, are not yet available.

Due to the methodological heterogeneity of diagnostic criteria and the exclusion of hospital studies in this research, generalizing evidence on the prevalence of CVDs is difficult. However, some research findings do suggest that the pooled prevalence of CVDs is quite high (24%-26%) among the Bangladeshi population [34, 35]. The average estimates are higher in Bangladesh than those of India (12%–27%) and Pakistan (around 20%) but considerably lower than those of Sri Lanka (31%–44%) [30].

Urbanization, an important underlying factor, was identified as the most common in all cases that have high prevalence of the CVD’s immediate risk factors. It is not possible to directly assess the effect of urbanization on the individual risk factors due to the lack of longitudinal data. However, the overall rural-urban distribution of CVDs risk factor prevalence raises issues which need greater attention. The urban population in Bangladesh has been growing on an average of 3.5% per year [36]; which is associated with unplanned industrialization [37]. The living standard of most of the cities has been reported to be falling [36] and the urban areas are experiencing a decline in air quality [38]. This kind of urbanization may also be conceived to be associated with unhealthy diet and less physical activity, leading to excessive weight gain, and, also, with increased stress and anxiety which, directly or indirectly, may affect other behavioral factors such as smoking [39].

### Strengths and limitations

Most of the studies on CVD risk factors have been conducted in and around Dhaka division and a few were in Sylhet, Chittagong and Rajshahi, four out of the seven divisions in Bangladesh. Thus, the findings may not be nationally representative. The urban data samples were not representative enough as participants had common environments (i.e., health care professionals, government employees, slum dwellers) [40–42] that were crucial. Moreover, the rural samples were demographically diverse yet geographically limited to particular villages, and therefore were not representative of the thousands of other villages of the country. Most of the large scale studies, with an adequate number of samples, have been conducted by non-government organizations with some international collaboration. This can result in biases in population selection and recruitment. Probable overestimation or underestimation of health conditions prevailed as samples were taken depending on the availability of laboratory facilities and data were taken from surveys based on self-reported information or medical examinations conducted for self-reported diseases. It was evident that high heterogeneity rose from the study quality and participants’ inclusion criteria that may have resulted in relatively weaker conclusions.

The main strength of our study is that we were able to include all the eligible studies through comprehensive search of the international and local databases without any language restriction. We used a comprehensive 'quality scoring instrument' to assess the quality of the included studies to incorporate in the analyses. We had large number of studies that reported prevalence separately for urban/rural and male/female which facilitated comparisons across the groups.

### Recommendations / further research areas

This study provides paradoxical findings i.e., both obesity and smoking do not differ between urban and rural areas as rest of the risk factors do. Larger scale studies with appropriate design (e.g., prospective cohort studies) are required to resolve this paradox. To date, no study has reported the impact of socioeconomic, environmental, nutritional and lifestyle factors affecting the CVDs and its immediate risk factors. In the past 20 years, substantial socioeconomic and demographic change has occurred in Bangladesh, and the economy has shifted gradually from a completely agricultural economy to one with a growing reliance on the industrial sector, resulting in an urbanizing effect on rural lifestyle [43]. Longitudinal studies are required to examine the impact of the sociodemographic and epidemiological transition on CVD risk. Nationally representative large scale studies with greater accuracy in the estimation of such kind of changes along with the precise subcomponents and assessment of their contribution on individual CVD risk factors will greatly assist in improving policy and program planning in the public health sector of Bangladesh.

## Conclusions

The prevalence of risk factors of CVDs, namely T2DM, HTN, dyslipidemia, obesity and smoking are quite high in Bangladesh and are generally higher than in the neighbouring countries. The ageing of the population is a very likely factor but urbanization may also have an influence on CVD risk through changes in dietary pattern and physical activity. Further research, in particular longitudinal studies, may help greatly in understanding the complex interaction of these factors. These studies may help designing policies and programs for the management and prevention of CVDs in Bangladesh.

## Supporting Information

S1 PRISMA Checklist (DOC)

S1 Fig Distribution of the research studies according to division in Bangladesh. (TIFF)

S2 Fig Prevalence of obesity in Bangladesh. (TIFF)

S1 Table Summary of studies reporting prevalence of diabetes mellitus in Bangladesh. (DOC)

S2 Table Summary of studies reporting prevalence of hypertension in Bangladesh. (DOC)

S3 Table Summary of studies reporting prevalence of dyslipidemia in Bangladesh. (DOC)

S4 Table Summary of studies reporting prevalence of smoking in Bangladesh. (DOC)

S5 Table Summary of studies reporting prevalence of CVDs in Bangladesh. (DOC)

S6 Table Summary of studies reporting prevalence of obesity and overweight in Bangladesh. (DOC)
