# Supplementary figures and images for: Prevalence of Risk Factors for Cardiovascular Diseases in Bangladesh: A Systematic Review and Meta-Analysis
Source: PLoS One. 2016 Aug 5;11(8):e0160180. doi: 10.1371/journal.pone.0160180 (PMC4975457; doi:10.1371/journal.pone.0160180)

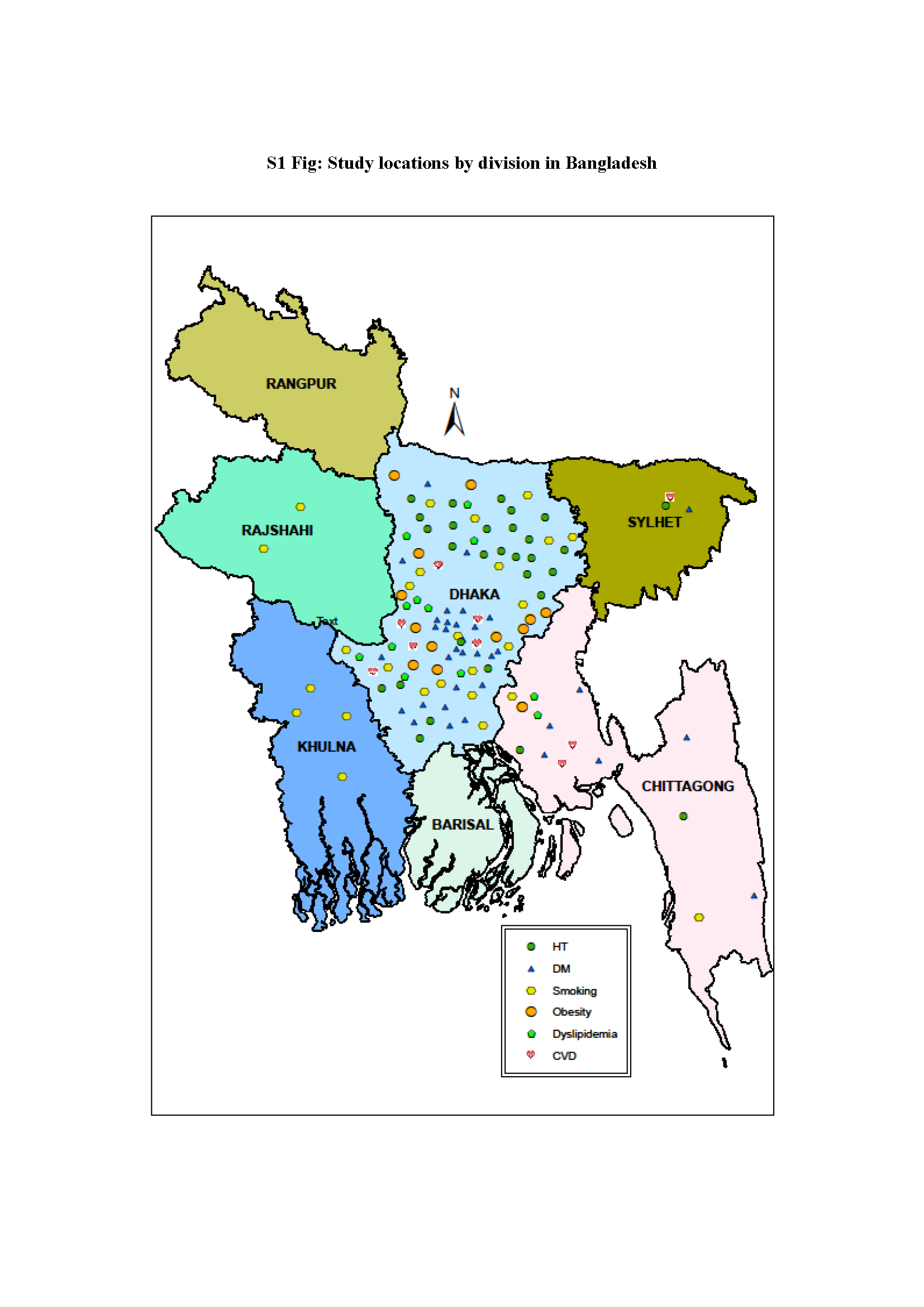

Supplement: S1 Fig — (TIFF) [file pone.0160180.s002.tiff]

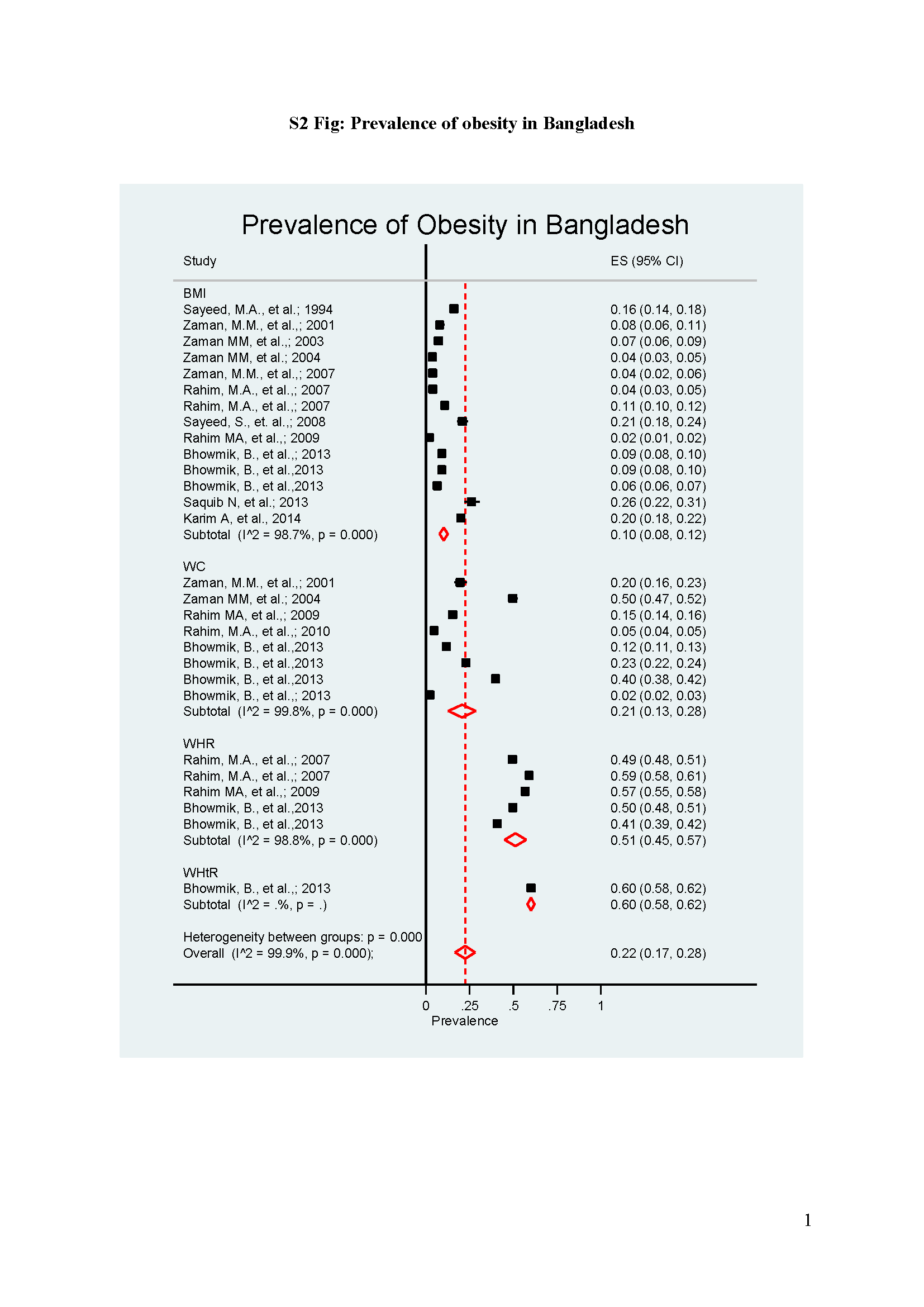

Supplement: S2 Fig — (TIFF) [file pone.0160180.s003.tiff]
