# Supplementary material for: Prevalence of Risk Factors for Cardiovascular Diseases in Bangladesh: A Systematic Review and Meta-Analysis
Source: PLoS One. 2016 Aug 5;11(8):e0160180. doi: 10.1371/journal.pone.0160180 (PMC4975457; doi:10.1371/journal.pone.0160180)
Supplement: S1 Table — (DOC) [file pone.0160180.s004.doc]

**S1 Table: Summary of studies reporting prevalence of diabetes mellitus in Bangladesh**

| **Author & year** | **Study design** | **Sample size, study place and data collection year** | **Sample characteristics** | **Diagnostic criteria** | **Prevalence** | | **Prevalence by strata** | **Significant risk factors** |
| --- | --- | --- | --- | --- | --- | --- | --- | --- |
| West KM., et al.,; 1966[1] | Cross-sectional | Total: 2049; East Pakistan: 519 Dhaka; Parpara, Amrailcherra, Comilla, Ghoira Study period: not mentioned | Urban and Rural | IGT ≥149mg/100ml | T2DM: 1.5% | | Male = 1.2%, Female = 2.8% | not reported |
| Mahtab, H., et al. 1983[2] | Cross-sectional | Total: 4980 Jurain, Lionhati Study period: April-June, 1982 | Semi Urban (Jurain), Rural (Lionhati), age ≥ 15 |  | T2DM = 0.7% | | Not reported | physical activity level |
| Sayeed, M.A., et al.; 1994 [3] | Cross-sectional | Total: 1005 Doharthana Study period: November 1992 | Rural, age≥15 | T2DM: FBG > 6.7 and 2-hBG >11.1; IGT: FBG ≥ 6.7 and 2-hBG7.8-11.1 | T2DM = 2.1%; IGT= 13.3% | | Among IGT cases Male = 17.6% and Female = 13.7% | increased age, high BMI, HTN |
| Sayeed, M.A., et al.,; 1995[4] | Cross-sectional | Total: 1005, Doharthana, Study period: Not mentioned | Rural, age >15 | T2DM: FBG > 6.7 and 2-hBG >11.1; IGT: FBG ≥ 6.7 and 2-hBG7.8-11.1 | T2DM = 2.1% IGT = 13.3% | | T2DM: Men = 3.1% and Female = 1.3%; IGT: Men = 14.4% and Female = 12.4%; | age, BMI |
| Sayeed, M.A., et al.,; 1997 [5] | Cross-sectional | Total: 6847 outside Dhaka Municiple corporation ;  Study period: not mentioned | Semi-urban, age ≥15 | 2hBG ≥ 11.0 | T2DM = 4.05% IGT = 7.5% | | T2DM: Male = 4.1% and Female = 3.9% | age BMI, height |
| Sayeed, M.A., et al.,; 1997[6] | Cross-sectional | Total: 2371 Kharua Union & Dhaka, Study Period: Not mentioned | Rural and Urban, age ≥ 20, pregnant and withmedication excluded | FBG ≥ 7.0 and/or 2-hBG≥11.0 | T2DM = 5.2% IGT = 8.0% | | T2DM: Men = 5.5% and Women = 4.8%; IGT:Men = 6.4% and Women = 10.5% | age, higher socioeconomic class, higher WHR |
| Zaman, M.M., et al.,; 2001[7] | Cross-sectional | Total: 240 Tetul jhora union, Savar; Study Period: 1996 | Rural, age ≥18 | FBG ≥7.0 or medication | T2DM = 2.5% | | Male = 3.8% and Female = 1.5% | not reported |
| Sayeed, M.A., et al.,; 2003[8] | Cross-sectional | Total: 4923 Rural Study period: not mentioned | Rural, age ≥20 | T2DM: FPG ≥ 7.0 | T2DM = 3.2% | | Male: 5.5% and female = 3.6% | BMI, WHR, WHtR |
| Sayeed, M.A., et al.,; 2003[9] | Cross-sectional | Total: 4923 Mymensingh, Study period: Sept 1999 - March 2000 | Rural, age ≥20 | T2DM: FPG ≥ 7.0; IFG: FPG = 6.1-6.9 | T2DM = 4.3% IFG = 12.4% | | T2DM: male = 5.2% and Female = 3.4%; IFG: Male = 12.7% and Female = 12.1% | age, BMI, WHR, higher income, Family history of DM, physical inactivity |
| Sayeed, M.A., et al., 2004[10] | Cross-sectional | Total: 1287 Khagrachori hill tract, Study Period: 2002 | Tribal Rural, age ≥20 | FPG ≥ 7.0 | T2DM = 6.6% IFG = 8.5% | | T2DM: male = 8.0% and Female = 5.6%; IFG: Male = 6.7% and Female = 5.2% | age, WHR, higher income |
| Sayeed, M.A., et al.,; 2005[11] | Cross-sectional | Total: 147 Nadail sub-district Study period: Sep 1999 - March 2000 | Rural, pregnant women, age = 18-44 | FBG ≥ 6.0 and 2-hBG≥7.8 | GDM: FBG = 6.8% 2hBG = 8.2% | | Not applicable | not reported |
| Rahman, M., et al; 2007[12] | Cross-sectional | Total: 975 Gazipur (Urbanizing Rural!!); Study Period: Nov-Dec 2004 | Urbanizing Rural, age ≥20 | FBG ≥ 7.0 and/or 2-hBG≥11.0 | | T2DM = 8.5% IFG = 8.25% | Male = 9.4% and Female = 8.0% | age, BMI |
| Zaman, M.M., et al.,; 2007[13] | Cross-sectional | Total: 447 Ekhlaspur Center of Health (ECOS), Chandpur; Study period: 2001 | Rural, age ≥20 | FBG ≥ 7.0 | T2DM = 2.0% | | Male = 2.0% and Female = 2.1% | not reported |
| Sayeed, M.A., et al.,; 2007[14] | Cross-sectional | Total: 5265 Dhaka city, Study period: June2002-May2003 | Urban, age ≥20 | FBG ≥ 6.1 and/or 2-hBG≥11.1 | IFG: 5.9% T2DM = 11.2% | | Slum = 7.4% Non-slum = 13.4% | family histry, Income, age, sedentary lifestyle, BMI, WHR |
| *Rahim, M.A., et al.,; 2007[15] | Cross-sectional | Total: 8738 Chandra Study Period: 1999-2004 | Rural, age ≥20 | FBG ≥ 6.1 and/or 2-hBG≥11.1 | T2DM = 2.3% | | Male = 1.9% and Female = 2.5% | BMI< WHR, Waist girth |
| *Hussain, A., et al.,; 2007[16] | Cross-sectional | Total: 4757 Chandra Study Period: 1999 | Rural, age ≥ 20 | DM: FBG>6.1; IFG: FBG >5.6-6.0 | T2DM = 2.3%; IFG = 4.7% | | T2DM: Male = 1.9% & Female = 2.54%; IFG: Male = 4.7% & Female = 4.67% | age, gender, WHR |
| Van Minh H, et al; 2008 [17] | Cross-sectional | Total: 7153 INDEPTH Asian Sites are: HSID (4023) WATCH (2000) Matlab (2073) Study Period: 2005 | Rural, age 25-64 | Self-reported | HSID: T2DM = 2.8%, WATCH: T2DM = 8.2%; Matlab: T2DM = 3.5% | | HSID: T2DM: Male = 3.5% and Female = 3.1%; WATCH: T2DM: Male = 35.4% and Female = 40.9%; Matlab: T2DM: Male = 2.8% and Female = 4.2%; | age and gender |
| Sayeed, S., et. al.,; 2008 [18] | Cross-sectional | Total: 705 Urban Community, Dhaka Study period: Oct 2004-Feb 2005 | Urban, age ≥25 | FBG ≥ 6.1 | T2DM = 21.1% | | Male = 27.1% and Female = 18.0% | not reported |
| ICDDR,B Bulletin, 2009[19] | Cross-sectional | Total: 517 HDSS, Matlab Study Period: not mentioned | Rural, Age = 27-50 | Fasting: T2DM≥ 7.0, IFG: ≥5.8-<7.0; 2h_BG: T2DM >11.1, IGT: 7.8-11.1 | Fasting: T2DM = 1.7% IFG = 10.8%; 2h-BG: T2DM = 2.9% IGT = 8.7% | | Fasting: T2DM among male = 1.7% & Female = 1.7%; IFG among male = 10.9% & female = 10.8% ; 2h-BG: T2DM among male = 1.3% & Female = 4.2; IGT among male = 6.6 & female = 10.4% | not reported |
| Ahsan, S. A., et al., 2009 [20] | Cross-sectional | Total: 163 UGC Employees Study period: Jan 2007-Dec 2007 | Urban (UGC Employee, sample collected at BSMMU), Mean age = 44.8 | FBG ≥ 7.0 | T2DM = 12.3% | | Not reported | not reported |
| *Rahim MA, et al.,; 2009 [21] | Cross-sectional | Total: 3967 Rural Study period: 2004 | Rural, age ≥20 | DM: FBG ≥ 6.1 and/or 2-hBG≥11.0; IGT: FBG ≤ 5.5 and/or 2-hBG=7.8-11.1; IFG: FBG = 5.6-6.0 and/or 2-hBG<7.7; IFG+IGT: 5.6-6.0 and/or 2-hBG = 7.8-11.0 | IFG = 1.3% IGT = 2.0% IFG+IGT = 4.3% DM = 7.0% | | IFG: Men = 1.1% and Female = 1.5%; IGT: Men = 1.4% and Female = 2.4%; IFG+IGT: Men = 3.1% and Female = 5.0%; T2DM: Men = 7.5% and Female = 6.7% | not reported |
| *Rahim, M.A., et al.,; 2010 [22] | Cross-sectional | Total: 3981 Chandra under Gazipur district; Study period: in 2004 | Rural, age ≥20 | DM: FBG ≥ 6.1 and/or 2-hBG≥11.0; IGT: FBG ≤ 5.5 and/or 2-hBG=7.8-11.1; IFG: FBG = 5.6-6.0 and/or 2-hBG<7.7; IFG+IGT: 5.6-6.0 and/or 2-hBG = 7.8-11.0 | IFG = 1.3% IGT = 2.0% IFG+IGT = 4.3% T2DM = 7.0% | | IFG: Men = 1.1% and Female = 1.5%; IGT: Men = 1.4% and Female = 2.4%; IFG+IGT: Men = 3.1% and Female = 5.0%; T2DM: Men = 7.5% and Female = 6.7% | not reported |
| Parr, J. D., et al.,; 2011 [23] | Cross-sectional | Total: 8591 Health Demographic Surveillance System (HDSS) (Abhoynagar, Mirsharai, Kamalapur) Study Period: January-December, 2009 | Residing in HDSS surveillance area, age >25, both gender | Self-reported | 5.50% | | Urban = 7.9% Rural = 3.2% | not reported |
| Boffetta, P., et al.,; 2011 [24] | Cross-sectional | Total: 11149 Health Effect of Arsenic Longitudinal Study (HEALS); Study Period: early 2000-2002 | self-reported DM, BMI: measured or self-reported, age ≥15 | Self-reported | T2DM = 5.3% | | not reported | age, BMI |
| Ahasan, H.A.M.N., et al.,; 2011 [25] | Cross-sectional | Total: 1000 Dhaka Secretariat; Study period: December 2008 | Urban; age | FBG ≥ 6.1 and/or 2-hBG≥11.0; | FBG based T2DM = 12.3% 2AG based T2DM = 7.5% | | not reported | age, sedentary lifestyle, family history, BMI, SBP, total Chol, TG |
| Akter, A., et al.,; 2011 [26] | Cross-sectional | Total: 836 Thakurgaon Study period: not mentioned | Rural, age ≥ 25 | DM: FBG ≥ 6.1 and/or 2-hBG≥11.0; IFG+IGT: 5.6-6.0 and/or 2-hBG = 7.8-11.0 | T2DM= 7.2% IGT+IFG = 6.5% | | T2DM: Male = 6.8% and Female = 7.6%; IFG+IGT: Male = 5.5% and Female = 7.6% | BMI, WHR |
| Rahim, M.A., et al., ; 2004 [27] | Cross-sectional | Total: 1555 Gazipur and Tangail, Study period: July-Aug 2001 | Urban Slum, age >20 | FBG ≥6.1 | T2DM = 8.1% | | T2DM for men = 7.7% and Women = 8.5% | age, sex, WHR |
| Das, M., et al., 2012 [28] | Cross-sectional | Total: 254 Dhaka Study period: January-June 2011 | Urban, age = 35-60 | DM: FBG ≥ 6.1 and/or 2-hBG≥11.0; IFG+IGT: 5.6-6.0 and/or 2-hBG = 7.8-11.0 | T2DM = 13.% | | T2DM for men = 13.0% and Women = 13.9% | not reported |
| Cravedi, P., et al.,; 2012 [29] | Cross-sectional | Total: 1518 Mollargaon, Sylhet Study Period: 2005-2008 | Rural, age >18, high risk for HTN, T2DM, prior CKD or Heart attack or stroke | by clinical staff | DM = 6.3% | | Not reported | not reported |
| *Bhowmik, B., et al.,; 2013[30] | Cross-sectional | Total: 2293 Chandra Study Period: in 2009 | Rural, age ≥ 20, | DM: FBG ≥ 7.0 and/or 2-hBG≥11.0; IGT: FBG < 7.0 and/or 2-hBG≥7.8-11.1; IFG: FBG ≥ 6.1-<7.0 and/or 2-hBG<7.8 | DM= 7.9% Pre-DM = 8.6% | | DM: Men = 3.5% Women = 4.4% Pre-DM: Men = 3.7% Women = 4.9% | not reported |
| *Bhowmik, B., et al.,2013 [31] | Cross-sectional | Total: 4757 Chandra (1999) Study period: 1999-2009 | Rural, age ≥ 20, | DM: FBG ≥ 7.0, Insulin or oral medicine, self-reported; IFG: FBG ≥ 6.1-<7.0 | In 1999: DM = 2.3%, IFG: 4.6%; in 2004: DM = 6.8%, IFG: 5.8%; in 2009: DM = 7.9%, IFG: 5.3% | | not applicable (difficult to interprate) | age, WHR, SBP |
| *Bhowmik, B., et al.,; 2013[32] | Cross-sectional | Total: 2293 Chandra, Study period: March-Dec 2009 | Rural, age ≥ 20, | DM: FBG ≥ 7.0, Insulin or oral medicine, self-reported | T2DM = 7.9% | | Male = 9.1%, female = 7.2% | not reported |
| Saquib N, et al.; 2013[33] | Cross-sectional | Total: 402 Dhaka Study period: not mentioned | Urban, age ≥30 | DM: FBG ≥ 7.0 or HbA1c ≥ 6.5% or medicine | T2DM = 35.3% | | Not reported | not reported |
| Akter S, et al.,; 2014[34] | Cross-sectional | Total: 7541 Nationally as BDHS data, Study period: 2011 | Urban & Rural, age ≥35 | DM: FBG ≥ 7.0 or medicine | DM = 9.7% Pre-DM=22.4% | | Urban: DM = 15.2% & pre-DM = 19.0%; Rural: DM = 8.3% & pre-DM = 23.5% | Richest household |
| Karim A, et al., 2014[35] | Cross-sectional | Total: 1134Bangaon union, Savar Study period: August 2006-April 2009 | Rural, age =18-65 | DM: FBG ≥ 7.0 or medicine | DM=7.1% | | Not reported | not reported |

* Data used from same source.

#Detail references are available at the end of S6 Table
