# Supplementary material for: Prevalence of Risk Factors for Cardiovascular Diseases in Bangladesh: A Systematic Review and Meta-Analysis
Source: PLoS One. 2016 Aug 5;11(8):e0160180. doi: 10.1371/journal.pone.0160180 (PMC4975457; doi:10.1371/journal.pone.0160180)
Supplement: S2 Table — (DOC) [file pone.0160180.s005.doc]

**S2 Table: Summary of studies reporting prevalence of hypertension in Bangladesh**

| **Author & year** | **Study design** | **Sample size, study place and data collection year** | **Sample characteristics** | **Diagnostic criteria** | **Prevalence** | **Prevalence by strata** | **Significant risk factors** |
| --- | --- | --- | --- | --- | --- | --- | --- |
| Malik, A., 1976[36] | Cross-sectional | Mothijhil, Kanchan, Dhaka Medical College, Mitford; Study period: not mentioned | Urban & Rural, age = 15-74 | not mentioned | HTN = 1.1% | not reported | not mentioned |
| Ullah W.; 1976[37] | Cross-sectional | Total: 17569 Agriculture uni, Medical college, Different jute mills Mymensingh medical college Study period: 1971-1975 | Rural, age ≥20 | not mentioned | HTN = 2.6% | Agriculture uni = 2.68% Medical college = 2.45% Different jute mills = 1.71% Mymensingh medical college = 2.36% | not mentioned |
| Islam, N., et al.,; 1979[38] | Cross-sectional | Total: 8172 Study place: Staff of Bangladesh secretariat, Dhaka Study period: Not mentioned | Urban, age ≥18, antihypertensive medication | DBP ≥90 | HTN=13.3% | not reported | not mentioned |
| Islam, N., et al.,; 1983[39] | Cross-sectional | Total: 5026 Village (place not mentioned) Study period: not mentioned | Rural | DBP ≥90 | HTN = 6.70% | not reported | not reported |
| Sayeed, M.A., et al.; 1994 [3] | Cross-sectional | Total: 1005 Dohar thana Study period: November 1992 | Rural, age≥15 | SBP≥140 and DBP ≥90 | sHTN= 10.5% dHTN = 9.0% | not reported | Increased age, high BMI, hyperglycemia |
| Sayeed, M.A., et al.,; 1995[4] | Cross-sectional | Total: 1005, Dohar thana, Study period: Not mentioned | Rural, age >15 | SBP≥140 and DBP ≥90 | sHTN = 10.5% dHTN = 9.0 | not reported | age, BMI, 2hBG |
| Abu Sayeed, M., et al.; 1998[40] | Cross-sectional | Total: 693 BIRDEM, Rural: 174 and Urban: 519; Male: 295 and Female:398 Study period: not mentioned | Rural (174), Urban (519); non-smokers, age 30-60 | SBP≥140 and DBP ≥90 | sHTN = 23.3% dHTN = 13.6% | Rural: sHTN = 18.4% and dHTN = 5.7%; Urban: sHTN = 24.9% and dHTN = 16.2%; Male: sHTN = 19.3% and dHTN = 11.2%; Female: sHTN = 26.1% and dHTN = 15.3% | age, overall obesity |
| Zaman, M.M., et al.,; 2001[7] | Cross-sectional | Total: 515 Tetuljhora union, Savar; Study Period: 1996 | Rural, age ≥18 | SBP≥140 and DBP ≥90 or medication *SBP≥160 and DBP ≥95 or medication | HTN senario 1: 12.9%; HTN senario 2: 5.8% | HTN senario 1: Male = 9.8% and Female = 15.6%; HTN senario 2: Male = 4.9% and Female = 6.5% | not reported |
| Hypertension study group; 2001 [41] | Cross-sectional | Total 480, Mymensingh metro-politon area and Muktagacha Thana, Study period: Dec 1999 to Feb 2000 | Urban and rural, age ≥60 | SBP≥140 and/or DBP ≥90 and/or medication | HTN = 64% | Urban = 75% and Rural = 53% | BMI, Education, DM, Urban |
| Sayeed, M.A., et al.,; 2002[42] | Cross-sectional | Total: 2361 Dhaka and karua union under nandail upazila of Mymenshingh; Study period: Oct 1995-March 1996 | Urban and rural, age ≥20 | SBP≥140 and DBP ≥90 | sHTN = 14.4% dHTN = 9.1% | Urban: Male sHTN = 11.4% and female sHTN = 9.0%; Male dHTN = 9.0% and female dHTN = 7.5%; Rural: Male sHTN = 21.4% and female sHTN = 12.5%; Male dHTN = 11.1% and female dHTN = 9.6% | age, BMI, rural area, upper class |
| Sayeed, M.A., et al.,; 2003 [9] | Cross-sectional | Total: 4923 Rural Study period: Sep 1999 - March 2000 | Rural, age ≥20 | T2DM: FPG ≥ 7.0 | T2DM = 3.2% | Male: 15.7% & female = 22.5% | BMI, WHR, WHtR |
| Zaman MM, et al.; 2004 [43] | Cross-sectional | Total: 1271 Ekhlashpur, Matlab; Study period: Sept 1999-Aug 2001 | Rural, age≥20 | SBP≥140 and/or DBP ≥90 and/or medication | HTN = 17.8% | Male = 17.3% & Female = 18.2% | not reported |
| Sayeed, M.A., et al.,; 2005[11] | Cross-sectional | Total: 147 Nandail sub district Study period: Sep 1999 - March 2000 | Rural, pregnant women, age = 18-44 | SBP≥140 or DBP ≥90 | sHTN = 6.8% dHTN = 5.4% | Not applicable | not reported |
| Chen, Y., et al.; 2006[44] | Cross-sectional | Total : 11116 HEALS study Study period: Oct 2000-May 2002 | Rural, age ≥18 | SBP≥140 or DBP ≥90 or medication | HTN = 13.3% | Male = 13.6% & Female = 13.0% | animal protein, dietary pattern |
| Zaman, M.M., et al.,; 2007[13] | Cross-sectional | Total: 447 Ekhlaspur Center of Health (ECOS), Chandpur; Study period: 2001 | Rural, age ≥20 | HTN: SBP≥140 and DBP ≥90 | HTN: 18.6% | men = 16.7% and women = 19.5% | not reported |
| Rahim, M.A., et al.,; 2007[15] | Cross-sectional | Total: 8738 Chandra Study Period: 1999-2004 | Rural, age ≥20 | SBP≥140 and DBP ≥90 | 1999: sHTN = 6.8%, dHTN = 6.6%; 2004: sHTN = 8.5%, dHTN = 6.9% | not reported | not reported |
| Ahmed, S., et al.,; 2007[45] | Cross-sectional | Total: 226 Sreepur thana Study period: April 2007 | Rural, age ≥50 | SBP≥135 and DBP ≥85 | sHTN = 17.7% dHTN = 16.8% | Male sHTN = 15.7% & Female sHTN = 20.0% | not reported |
| Van Minh H, et al; 2008[17] | Cross-sectional | Total: 7153 INDEPTH Asian Sites are: HSID (4023) WATCH (2000) Matlab (2073) Study Period: 2005 | Rural, age 25-64 | Self-reported | HTN at HSID = 14.6%, HTN at WATCH = 17.1%; HTN at Matlab = 10.6% | HTN at HSID: Male = 10.4% and Female = 18.9%; HTN at WATCH: Male = 9.9% and Female = 24.2%; HTN at Matlab: Male = 6.7% and Female = 14.6%; | age and gender |
| Sayeed, S., et. al.,; 2008[18] | Cross-sectional | Total: 705 Urban Community, Dhaka Study period: Oct 2004-Feb 2005 | Urban, age ≥25 | SBP≥135 and DBP ≥85 | sHTN = 14.7% dHTN = 22.2% | Male sHTN = 15.9% &dHTN = 27.5% Female sHTN = 14.1% &dHTN = 19.5% | not reported |
| Ahsan, S. A., et al., 2009[20] | Cross-sectional | Total: 163 UGC Employees Study period: Jan 2007-Dec 2007 | Urban (UGC Employee, sample collected at BSMMU), Mean age = 44.8 | SBP≥140 or DBP ≥90 | HTN: 16.6% | Not reported | not reported |
| Hoang Van, M., et al., 2009 [46] | Cross-sectional | Total: 8069 Matlab, Mirsarai, Abhoynagar WATCH, Study Period: 2005 | Rural, age = 25-64 | SBP≥140 and DBP ≥90 | Senario 1: HTN = 16.9%; Senario 1: HTN = 9.8% | Senario 1 based place wise: Matlab = 17.1%, Mirsarai = 24.1%, Abhoynagar = 16.8% and WATCH = 9.3%; Gender: Men = 12.5%, 20.3%, 13.3% and 7.4%; Women = 21.0%, 27.4%, 19.8% and 11.2%; Senario 2 based place wise: Matlab = 9.1%, Mirsarai = 15.3%, Abhoynagar = 10.2% and WATCH = 4.5%; Gender: Men = 5.0%, 12.3%, 7.5% and 2.6%; Women = 12.4%, 18.0%, 12.6% and 6.3% | gender, overweight and obesity |
| Moni, MA., et al.; 2010 [47] | Cross-sectional | Total: 317 Dhaka city (U), Study Period: Jan-June 2006 | Urban (Nakhal para, badda and mirpur), Mean age = 67.1 | SBP≥140 and DBP≥or medication | 44.80% | Male = 53.5 and Female = 38.8 | overweight/obese or at health risk by WC/WHR |
| Das, S., et al., 2010[48] | Cross-sectional | Total: 1200 Dhaka medical college Study period: not mentioned | Urban, | Unknown | HTN = 17.3% | Unknown | unknown |
| Parr, J. D., et al.,; 2011[23] | Cross-sectional | Total: 8591 Health Demographic SurveillanceSystem (HDSS) (Abhoynagar, Mirsharai, Kamalapur) Study Period: January-December, 2009 | Residing in HDSS surveillance area, age >25, both gender | Self-reported | 13.60% | Urban = 16.1%; Rural = 12.4% | not reported |
| Islam, M.R., et al.; 2012 [49] | Cross-sectional | Total: 1004 Lakshamupazila, Comilla; Study period: July 2009 | Rural age≥30 | SBP≥140 and DBP ≥90 | HTN = 6.6 | Not reported | higher arsenic drinking water or duration, pulse pressure |
| Cravedi, P., et al.,; 2012[29] | Cross-sectional | Total: 1518 Mollargaon, Sylhet Study period: not mentioned | Rural, age >18, high risk for HTN, T2DM, prior CKD or Heart attack or stroke | by clinical staff | HTN = 18.5% | Not reported | not reported |
| Bhowmik, B., et al.,; 2013[30] | Cross-sectional | Total: 2293 Chandra Study Period: in 2009 | Rural, age ≥ 20, | Not reported | HTN = 15.50% | Not reported | not reported |
| Bhowmik, B., et al.,2013 [31] | Cross-sectional | Total: 4757 Chandra (1999) Study period: 1999-2009 | Rural, age ≥ 20, | SBP≥140 and DBP ≥90 | 1999: HTN = 14.3%; 2004: HTN = 18.4%; 2009: HTN = 14.0% | in 1999: Male = 13.2% and Female = 15.4%; in 2004: Male = 18.7% and Female = 18.2%; in 2009: Male = 16.8% and Female = 14.3%; | not reported |
| Bhowmik, B., et al.,; 2013[32] | Cross-sectional | Total: 2293 Chandra, Study period: March-Dec 2009 | Rural, age ≥ 20, | SBP≥140 and DBP ≥90 | HTN = 15.5% | Male = 17.5% Female = 14.3% | not reported |
| Karim A, et al., 2014[35] | Cross-sectional | Total: 1134  Bangaon union,Savar Study period: August 2006-April 2009 | Rural, age =18-65 | SBP≥140 and DBP ≥90 | HTN = 19.1% | Not reported | not reported |
| Ahmed A, et al.,; 2014 [50] | Cross-sectional | Total: 212  Araihazar Upazilla, Narayangong;  Study period: Sept-Dec 2009 | Rural, age >30 years | SBP≥140 and DBP ≥90 | sHTN = 15.6% dHTN = 12.3% | Not reported | age and BMI with sHTN |

Some denominator but numerator is diastolic BP and they define HTN separately using SBP and DBP, not both for same person

##Detail references are available at the end of S6 Table
