# Supplementary material for: Prevalence of Risk Factors for Cardiovascular Diseases in Bangladesh: A Systematic Review and Meta-Analysis
Source: PLoS One. 2016 Aug 5;11(8):e0160180. doi: 10.1371/journal.pone.0160180 (PMC4975457; doi:10.1371/journal.pone.0160180)
Supplement: S3 Table — (DOC) [file pone.0160180.s006.doc]

**S3 Table: Summary of studies reporting prevalence of dyslipidemia in Bangladesh**

| **Author & year** | **Study design** | **Sample size, study place and data collection year** | **Sample characteristics** | **Diagnostic criteria** | **Prevalence** | **Prevalence by strata** | **Significant risk factors** |
| --- | --- | --- | --- | --- | --- | --- | --- |
| MostafaZaman, M., et al.,; 2006 [51] | Cross-sectional | Total: 447 Matlab, Study period: 2001 | Rural, age = 20-79 | high cholesterol, >240mg/dl high LDL, >160 mg/dl Low HDL <40mg/dl high TG ≥200 mg/dl Total Chol/total HDLC (>5.5) | high cholesterol = 16.1% high LDL = 66.4% Low HDL = 20.4% high TG = 15.0% Total Chol/total HDLC = 32.7% | Male: high cholesterol = 15.9%, high LDL = 68.9%, Low HDL = 20.5%, high TG = 12.6% and Total Chol/total HDLC = 37.1%; Female: high cholesterol = 16.2%, high LDL = 65.2%, Low HDL = 20.3%, high TG = 16.2% and Total Chol/total HDLC = 30.3% | not reported |
| Shekhar HU, et al.; 2006 [52] | Cross-sectional | Total: 96 Dhaka; Study period: Nov 2003-Aug 2004 | Urban, age = 20-65 | Not reported | Hyper chol = 33%; Hyper TG = 42%; Low HDL-C = 64% | Not reported | Not reported |
| Sayeed, S., et. al.,; 2008 [18] | Cross-sectional | Total: 705 Urban Community, Dhaka Study period: Oct 2004-Feb 2005 | Urban, age ≥25 | High cholesterol, >200mg/dl Low HDL <40mg/dl High TG ≥150 mg/dl | High cholesterol = 36.3% Low HDL = 43.8% High TG = 45.1% | Male: High cholesterol = 34.3%; Low HDL = 45.2% High TG = 51.0% Female: High cholesterol = 37.4%; Low HDL = 43.1% High TG = 42.1% | not reported |
| Ahsan, S. A., et al., 2009 [20] | Cross-sectional | Total: 163 UGC Employees Study period: Jan 2007-Dec 2007 | Urban (UGC Employee, sample collected at BSMMU), Mean age = 44.8 | Not reported | Dyslipidemia = 20.9% | Not reported | not reported |
| Alam, MB., et al.,; 2009 [53] | Cross-sectional | Total: 1000 Employees of Bangladesh secretariat Study period: Dec 2008 | Employees of Bangladesh secretariat , Urban, age >18 | high cholesterol, >200mg/dl high LDL, >160 mg/dl Low HDL <40mg/dl high TG ≥200 mg/dl | high cholesterol = 17.3%; high LDL = 48.5%; Low HDL = 75.6%; high TG = 48.5% | Not reported | not reported |
| Parr, J. D., et al.,; 2011 [23] | Cross-sectional | Total: 8591 Health Demographic Surveillance System (HDSS) (Abhoynagar, Mirsharai, Kamalapur) Study Period: Jan-Dec, 2009 | Residing in HDSS surveillance area, age >25, both gender | Self-reported | Dyslipidemia = 4.8% | Urban = 5.0%; Rural = 0.1% | not reported |
| Bhowmik, B., et al.,; 2013 [30] | Cross-sectional | Total: 2293 Chandra Study Period: in 2009 | Rural, age ≥ 20, | Cut of value: Chol ≥ 5.0, TG ≥ 1.7, LDL ≥ 3.0, HDL-chol<1.03 for men and <1.29 for women; Dyslipidemia: TG ≥ 1.7, HDL <1.03 for men and <1.29 for women | Dyslipidemia = 28.7% | Total Chol = 4.5%; TG = 1.6%; HDL-chol = 0.86%; LDL-chol = 2.79% | not reported |
| Bhowmik, B., et al.,; 2013 [32] | Cross-sectional | Total: 2293 Chandra, Study period: March-Dec 2009 | Rural, age ≥ 20, | Dyslipidemia: TG ≥ 1.70, HDL <1.04 for men and <1.29 for women | Dyslipidemia = 28.7% | Male = 35.3% and Female = 24.8% | not reported |
| FariaA., et al.,; 2010 [54] | Cross-sectional | Total: 100 BIRDEM hospital Study time: not mentioned | Urban, age = 40-80 | not reported | High TG = 89%; Low LDL = 87% | not reported | not reported |
| Das SK et al.,; 2012[55] | Cross-sectional | Total: 51353 Clinical biochemistry laboratory data, ICDDR,B,; Study Period: 2005-2011 | Urban, age≥19, hospital laboratory data | Cut of value: Chol ≥ 4.44, TG ≥ 1.56, LDL ≥ 2.74, HDL-chol<1.11 mmol/l | High cholesterol = 1.3%; high LDL = 1.3%; Low HDL = 0.5%; high TG = 1.05% | not reported | age |
| Islam N et al.,; 2012 [56] | Cross-sectional | Total: 3201 Bhaluka upazilla, Mymensingh; Study Period: 2005-2011 | Rural, age≥18, | High cholesterol, >200mg/dl; high LDL, >160 mg/dl; Low HDL <40 mg/dl; high TG ≥200 mg/dl | High cholesterol = 51.3%; high LDL = 20.1%; Low HDL = 8.8%; high TG = 35.7% | not reported | not reported |

#Detail references are available at the end of S6 Table
