# Supplementary material for: Prevalence of Risk Factors for Cardiovascular Diseases in Bangladesh: A Systematic Review and Meta-Analysis
Source: PLoS One. 2016 Aug 5;11(8):e0160180. doi: 10.1371/journal.pone.0160180 (PMC4975457; doi:10.1371/journal.pone.0160180)
Supplement: S4 Table — (DOC) [file pone.0160180.s007.doc]

**S4 Table: Summary of studies reporting prevalence of smoking in Bangladesh**

| **Author & year** | **Study design** | **Sample size, study place and data collection year** | **Sample characteristics** | **Diagnostic criteria** | **Prevalence** | **Prevalence by strata** | **Significant risk factors** |
| --- | --- | --- | --- | --- | --- | --- | --- |
| Cohen N, et al., 1983[57] | Cross-sectional | Total: 989; 2 village of north-west of Bangladesh; study period: March-April 1980 | Rural, age <15 years, male and female | Answer for 1 or more cigarettes per day | Tobacco smoking = 34.4% | male = 71% and Female 1.0% |  |
| Islam N, et al., 1990[58] | Cross-sectional | Total: 2008; Bangladesh Secretariat Staff; study period: October 1989 | Urban, age <25 years, male and female | not mentioned | Tobacco smoking = 38.6% |  | age, level of education and employment status |
| Ahsan H, et al., 1998 [59] | Cross-sectional | Total: 617; school and slum dwellers; study period: 1991 | Urban, age 12-20 years, male | not clear | Tobacco smoking = 28.7% (school) and 67.8% (slum dwellers) | male = 71% and Female 1.0% |  |
| Zaman MM, et al., 2001 [7] | Cross-sectional | Total: 510; Tetulganj, Savar; study period: 1996 | Rural <18 years, male and female | Answer yes or no for the question of whether individual having smoking habit | Tobacco smoking = 28.1% | Male=50.3% and Female=2.9% |  |
| Khan MMH, et.al.; 2006 [60] | Cross-sectional | Total: 4297; Bangladesh (Bangladesh demographic and health survey) study period: 2004 | Rural and Urban BDHS 2004 data, only male, age 15-54 years | Answer yes or no for the question of having cigarettes, self-reported | Tobacco smoking = 49.6% | Urban = 41.1 and Rural = 23.6 | age , education, place of resident, marital status, having STDs, premarital and extra marital sex |
| Choudhury K, et al., 2007[61] | Cross-sectional | Total: 6464; Health and demography surveillance at Chakaria, cox's bazar district; study period: October 1994 | Rural, age <15 years, male and female | Answer yes or no for the question of whether individual having smoking habit | Tobacco smoking = 43.4% | male = 63.2% and Female 24.2% |  |
| Rahman M, et al., 2007 [62] | Cross-sectional | Total: 1000; Rickshaw puller in Dhaka city; study period: Aug-Oct 2003 | Urban, male | Answer yes or no for the question of whether individual having smoking habit | Tobacco smoking = 75.9% |  | older, poor schooling |
| Khan MMH, et al., 2009[63] | Cross-sectional | Total: 12155; Urban Health Survey; study period: 2006 | Urban slum and non-slum, male | Answer yes or no for the question of whether individual having smoking habit | Tobacco smoking = 53.6% | smoking prevalence among urban slum: Barisal - 57.9%, Chittagong - 58.0, Dhaka - 60.0, Khulna - 51.4, Rajshahi - 65.9 and Sylhet - 79.1; among urban non slum: Barisal - 38.6%, Chittagong - 46.3, Dhaka - 45.4, Khulna - 46.8, Rajshahi - 52.3 and Sylhet - 47.6 | marital status, birth place, religion |
| Ali A, et al., 2009 [64] | Cross-sectional | Total: 9208; Health and demographic surveillance System of The INDEPTH network (Matlab, Mirsarai, Abhoynagar and WATCH); study period: Not mentioned | Urban and Rural, male, age 25-64 years | Answer yes or no for the question of whether individual having smoking habit | Tobacco smoking: Matlab=52.5%, Mirsarai=62.6%, Abhoynagar=46.6% and WATCH=59.7% | Prevalence among women in Matlab=0.8%, Mirsarai=0.3%, Abhoynagar=1.4% and WATCH=2.7% | age, poor education |
| Mostafa MG, et al., 2008 [65] | Cross-sectional | Total: 5372; Anowara Diagnostic Center of Bangladesh; study period: 2003-2006 | Rural and Urban, age <18 years, male and female | Not mentioned | Tobacco smoking = 74.6% |  |  |
| Flora MS, et al., 2009[66] | Cross-sectional | Total: 35446;Mirpur (urban) and Kaliganj (rural); study period: 2001-2003 | Rural and Urban, age <20 years, male and female | Answer yes or no for the question of whether individual having smoking habit | Tobacco smoking = 20.5% | In Rural: Male=43.2% and Female=4.2%; In urban: Male = 41.1% and Female=0.3%; overall: male=42.2% and Female=2.3% | Socio-demographic variables |
| Siddiqui MNA, et al., 2011[67] | Cross-sectional | Total: 200; Rajshahi Islami Bank Medical College; study period: July 2010-march 2011 | Urban, male | not mentioned, self-reported | Tobacco smoking = 20.0% |  |  |
| Hypertension Study Group, 2001 [41] | Cross-sectional | Total: 2008; Mymensingh municipal corporation and Muktagachathana; study period: Dec 1999- Feb 2000 | Urban and Rural age ≥60 years, male and female | Answer yes or no for the question of who smoked any number of cigarette regularly | Tobacco smoking = 12.0% | Urban = 5.8 and Rural = 18.3 |  |
| Kamal SMM, et al., 2011[68] | Cross-sectional | Total: 3255; Bangladesh Demographic and Health Survey(BDHS) 2004; study period: 2004 | Rural and Urban, age <15 years, male and female | Answer yes or no for the question of whether individual having smoking habit | Tobacco smoking = 49.0% |  |  |
| Pesola GR, et al., 2011[69] | Cross-sectional | Total: 11746; Health Effects of Arsenic Longitudinal Study (HEALS) Araihazar; study period: Not mentioned | Rural <18 years, male and female | not mentioned | Tobacco smoking = 28.9% | Male=62.3% and Female=3.7% |  |
| Razzak A. 2011 [70] | Cross-sectional | Total: 364; Hospitalized infectious disease patient at Jhenaidaha and Kushtia; study period: Feb-March 2009 | Rural | not mentioned | Tobacco smoking = 21.2% |  |  |
| Hanifi SM, et al., 2011 [71] | Cross-sectional | Total: 26981; Health and demography serveillance at Chakaria, cox's bazar district; study period: October 1994 and 2008 | Rural, age <15 years, male and female | Answer yes or no for the question of whether individual having smoking habit | Tobacco smoking = 40.9% (in 1994), and 26.7%(in 2008) | In 1994: Male = 59.89% and Female 19.99%; In 2008: Male = 40.56% and Female 12.29% |  |
| Abdur R, et al., 2011 [72] | Cross-sectional | Total: 2000; Matlab Health and demography surveillance system; study period: 2005 | Rural, age 25-59 years, male and female | Answer yes or no for the question of whether individual having smoking habit | Tobacco smoking = 27.4% | male = 53.9% and Female 0.8% | tobacco consumption decrease with increase of education |
| Kamal SM, et al., 2011[68] | Cross-sectional | Total: 474; Islamic University, Kushtia; study period: March-May 2009 | Rural, age <17 years, male | Answer yes or no for the question of whether individual having smoking habit | Tobacco smoking = 36.1% |  |  |
| Palipudi KM, et al., 2012 [73] | Cross-sectional | Total: 9629; Global Adult Tobacco Survey (GATS); study period: 2008-2010 | Rural and Urban, age <15 years, male and female | Answer yes or no for the question of having smoking habit | Tobacco smoking = 43.3% | male = 58.0% and Female 28.7% | education level, wealth index, all social determinants |
| Sinha DN, et. al,; 2012[74] | Cross-sectional | Total: 1000; the global health professional student survey, Bangladesh Study period: 2005-2008, data collection 2006 | national or city level, youth in age | Answered 1 or more days to the question on how many days did you smoke cigarettes, self-reported | current smoker = 25.5% | male = 46.5% (233/500) and Female 4.4% (22/500) | not mentioned |
| Kishore J, et al., 2013 [75] | Cross-sectional | Total: 3651921; Global Adults Tobacco Survey (GATS); study period: 2009 | Rural and Urban, age <15 years, male and female | Answer yes or no for the question of whether individual having smoking habit | Tobacco smoking = 18.3% | Male=3.44% and Female=0.21% in millions a | age, gender, occupation and wealth index |
| Kabir MA, et al., 2013[76] | Cross-sectional | Total: 1576; slum dwellers (Urban Health Survey) study period: 2006 |  | Answer yes or no for the question of having smoking habit currently, self-reported | current smoker = 42.3% | smoking cigarettes = 41.1% and Bidis = 3.1% | age, marital status, education, duration of living in slums, STDs |
| Kabir MA, et al., 2013[77] | Cross-sectional | Total: 3771; Bangladesh (Bangladesh demographic and health survey) study period: 2007 | Urban and Rural BDHS 2007 data, only male, age 15-54 years | Answer yes or no for the question of having cigarettes, self-reported | Tobacco smoking = 60.0% | Urban = 64.0 (/857)and Rural = 71.9 (/2913) | illegal drug use 4 times higher among smokers |
| Bartlett E, et al., 2013[78] | Cross-sectional | Total: 32665; HDSS surveillance area (abhoynagar 11593, Mirsarai 11994 and -Kamlapur 9078);  study period: 2009 | Rural and Urban; age 25 years, male and female, | number of packs per day per year, self-reported | Tobacco smoking = 26.8% | Urban = 29.6 and Rural = 25.7 | not mentioned |
| Sreeramareddy CT, et al., 2014[79] | Cross-sectional | Total: 3771; demographic and health survey; study period: March - August 2007 | Rural, age <15 years, male | Answer yes or no for the question of whether individual having smoking habit | Tobacco smoking = 60.0% |  | higher age, lower education, poverty |

#Detail references are available at the end of S6 Table
