# Supplementary material for: Prevalence of Risk Factors for Cardiovascular Diseases in Bangladesh: A Systematic Review and Meta-Analysis
Source: PLoS One. 2016 Aug 5;11(8):e0160180. doi: 10.1371/journal.pone.0160180 (PMC4975457; doi:10.1371/journal.pone.0160180)
Supplement: S5 Table — (DOC) [file pone.0160180.s008.doc]

**S5 Table: Summary of studies reporting prevalence of CVDs in Bangladesh**

| **Author & year** | **Study design** | **Sample size, study place and data collection year** | **Sample characteristics** | **Diagnostic criteria** | **Prevalence** | **Prevalence by strata** | **Significant risk factors** |
| --- | --- | --- | --- | --- | --- | --- | --- |
| Malik, A., 1976[36] | Cross-sectional | Total: 7062 Mothijhil, Kanchan, Dhaka Medical College, Mitford; Study period: not mentioned | Urban & Rural, age = 15-74 | X ray of chest, Electrocardiogram | CVD = 2.9%, IHD = 0.33% | IHD: Male = 22 and Female = 2 | HTN |
| Abu Sayeed, M., et al.; 1998 [80] | Cross-sectional | Total: 693 BIRDEM, Rural: 174 and Urban: 519; Male: 295 and Female:398 Study period: not mentioned | Rural (174), Urban (519); non-smokers, age 30-60 | (a) history of angina plus ECG-positive either on rest or on stress; and (b) post-myocardial infarction (MI) with Q-wave MI or non-Q-MI or echocardiographic evidence(s) | CHD = 18.6%; CHD at Rural = 17.8% and CHD at Urban = 18.9% | Men - 16.6%, Women - 20.1%; Rural - 17.8% and Urban 18.9% | increased age, central obesity (WHR), HTN |
| Zaman, M.M., et al.,; 2007[13] | Cross-sectional | Total: 447 Ekhlaspur Center of Health (ECOS), Chandpur; Study period: 2001 | Rural, age ≥20 | IHD = pathological Q wave or current medication | IHD = 3.4% | IHD: Male = 4.7% and Female = 2.7% | not reported |
| Ahmed, S., et al.,; 2007[45] | Cross-sectional | Total: 226 Sreepurthana Study period: April 2007 | Rural, age ≥50 | IHD not defined | IHD = 2.7% | Not reported | not reported |
| Van Minh H, et al; 2008[17] | Cross-sectional | Total: 7153 INDEPTH Asian Sites are: HSID (4023) WATCH (2000) Matlab (2073) Study Period: 2005 | Rural, age 25-64 | Self-reported | 14.10% | In HSID: Stroke: Male = 2.0% and Female = 1.5%; Heart Disease: Male = 6.4% and Female = 7.8%; In WATCH: Stroke: Male = 0.5% and Female = 0.7%; Heart Disease: Male = 66.3% and Female = 77.7%; In Matlab: Stroke: Male = 1.6% and Female = 1.8%; Heart Disease: Male = 5.3% and Female = 10.7%; | age and gender |
| Ahsan, S. A., et al., 2009[20] | Cross-sectional | Total: 163 UGC Employees Study period: Jan 2007-Dec 2007 | Urban (UGC Employee, sample collected at BSMMU), Mean age = 44.8 | IHD not defined | IHD = 19.6% | Not reported | not reported |
| Sayeed MA, et al.; 2010 [[80](#_ENREF_45)] | Cross-sectional | Total: 768 Nandail sub-district of Mymensingh Study period: 2002-2003 | Rural, age ≥20 | (a) history of angina plus ECG-positive either on rest or on stress; and (b) post-myocardial infarction (MI) with Q-wave MI or non-Q-MI © diagnosed by a cardiologist | CHD = 1.58% | Male = 1.5% and female = 2.0% | age, sex, social class and obesity, higher 2hBG (≥7.0), higher ACR (≥17.2), family history |
| Parr, J. D., et al.,; 2011[23] | Cross-sectional | Total: 8591 Health Demographic Surveillance System (HDSS) (Abhoynagar, Mirsharai, Kamalapur) Study Period: January-December, 2009 | Residing in HDSS surveillance area, age >25, both gender | Self-reported | CHD = 5.1% Stroke = 2.0% | CHD: Urban = 6.0%, Rural = 4.7%; for Stroke: Urban = 1.8%, Rural = 1.9% | not reported |
| Mohammad, Q.D., et al.,; 2011 [81] | Cross-sectional | Total: 15627 Dhaka, Gazipur, Mymensingh; Study period: June 2001-May 2003 | Urban, Semi-urban and Rural, age ≥40 | a team of senior neurologist by full examination | Stroke = 0.3% | Urban = 0.31%, Sub-urban = 0.231% and Rural = 0.327% | not reported |
| Cravedi, P., et al.,; 2012[29] | Cross-sectional | Total: 1518 Mollargaon, Sylhet Study period: not mentioned | Rural, age >18, high risk for HTN, T2DM, prior CKD or Heart attack or stroke | by clinical staff | MI/CVD = 0.7% | Not reported | not reported |

# Detail references are available at the end of S6 Table
