# Supplementary material for: Prevalence of Risk Factors for Cardiovascular Diseases in Bangladesh: A Systematic Review and Meta-Analysis
Source: PLoS One. 2016 Aug 5;11(8):e0160180. doi: 10.1371/journal.pone.0160180 (PMC4975457; doi:10.1371/journal.pone.0160180)
Supplement: S6 Table — (DOC) [file pone.0160180.s009.doc]

**S6 Table: Summary of studies reporting prevalence of obesity and overweight in Bangladesh**

| **Author & year** | **Study design** | **Sample size, study place and data collection year** | **Sample characteristics** | **Diagnostic criteria** | **Prevalence** | **Prevalence by strata** | **Significant risk factors** |
| --- | --- | --- | --- | --- | --- | --- | --- |
| Sayeed, M.A., et al.; 1994 [3] | Cross-sectional | Total: 1005 Doharthana Study period: November 1992 | Rural, age ≥15 | ≥23.0 BMI | BMI - 16.02% | Not mentioned | not mentioned |
| Zaman, M.M., et al.,; 2001 [7] | Cross-sectional | Total: 515 Tetuljhoraunion,Savar; Study Period: 1996 | Rural, age ≥18 | BMI ≥25.0 kg/m2 WC ≥94 (men) and 80 cm (women) | 7.8% 10.2% | BMI: Male = 6.8% Female = 8.8%; WC: Male = 2.9% Female 16.8% | not mentioned |
| Zaman MM, et al.,; 2003[82] | Cross-sectional | Total: 896 Savar, Study period: 1998 | Rura, age = 20-69 | Overweight: BMI ≥25.0 kg/m2 | BMI = 7.1% | BMI: Male = 3.5% & Female = 9.9% | not reporetd |
| Zaman MM, et al.; 2004 [43] | Cross-sectional | Total: 1271 Ekhlashpur, matlab; Study period: Sept 1999-Aug 2001 | Rural, age≥20 | Obesity: BMI ≥25.0 kg/m2 WC ≥94 (men) and 80 cm (women) | BMI = 3.6% WC = 7.9% | BMI: Male = 2.4% & Female = 4.3%; WC: Male = 1.7% & Female = 11.4% | not reported |
| Zaman, M.M., et al.,; 2007[13] | Cross-sectional | Total: 447 Ekhlaspur Center of Health (ECOS), Chandpur; Study period: 2001 | Rural, age ≥20 | Overweight: BMI≥25.0 | high BMI = 3.9% | Overweight: Men = 2.0% and women = 4.8% | not reported |
| Rahim, M.A., et al.,; 2007[15] | Cross-sectional | Total: 8738 Chandra Study Period: 1999-2004 | Rural, age ≥20 | Overweight: BMI≥25.0, WHR >0.9 (men) and >0.8 (women) | in 1999: BMI = 10.7%, WHR = 59.1; in 2004: BMI = 4.0%, WHR = 45.7 | in 1999: WHR: male = 14.8% & Female = 68.9%; in 2004: WHR: male = 30.2% & Female = 63.7% | not reported |
| Sayeed, S., et. al.,; 2008[18] | Cross-sectional | Total: 705 Urban Community, Dhaka Study period: Oct 2004-Feb 2005 | Urban, age ≥25 | Obesity: BMI ≥25.0 kg/m2 | BMI = 20.9% | BMI: Male = 10.1% & Female = 26.4% | not reported |
| Rahim MA, et al.,; 2009[21] | Cross-sectional | Total: 3967 Rural Study period: 2004 | Rural, age ≥20 | Obesity: BMI (>25kg/m2), WC (men ≥ 90 cm and female ≥ 80 cm), WHR (men ≥ 0.90 and female ≥ 0.80) | BMI = 10.7% WC = 15.9% WHR = 59.3% | BMI: male = 3.9%, Female = 5.6%; WC: male = 5.8%, Female = 8.4%; WHR: male = 23.5%, Female = 34.0% |  |
| Rahim, M.A., et al.,; 2010[22] | Cross-sectional | Total: 3981 Chandra under Gazipur district;  Study period: in 2004 | Rural, age ≥20 | Not Reported | BMI = 10.3%, WC = 15.2%, WHR = 57.3% | not reported | not reported |
| FariaAet al; 2010[54] | Cross-sectional | Total: 100 BIRDEM hospital Study time: not mentioned | Urban, age = 40-80 | not reported | WC = 67% | not reported | not reported |
| Parr, J. D., et al.,; 2011 [23] | Cross-sectional | Total: 8591 Health Demographic Surveillance System (HDSS) (Abhoynagar, Mirsharai, Kamalapur) Study Period: January-December, 2009 | Residing in HDSS surveillance area, age >25, both gender | Self-reported | Obesity: 10.3% | Urban = 7.2% and Rural = 0.2% | not reported |
| Bhowmik, B., et al.,; 2013[30] | Cross-sectional | Total: 2293 Chandra Study Period: in 2009 | Rural, age ≥ 20, | BMI (>25kg/m2), WC (men ≥ 90 cm and female ≥ 80 cm), WHR (not mentioned) | BMI = 26.2%, WC = 39.8%, WHR =71.6% | not reported | not reported |
| Bhowmik, B., et al.,2013[31] | Cross-sectional | Total: 4757 Chandra (1999) Study period: 1999-2009 | Rural, age ≥ 20, | BMI (>25kg/m2), WC (men ≥ 90 cm and female ≥ 80 cm), WHR (men ≥ 0.90 and female ≥ 0.80) | in 1999: BMI = 4.4%, WHR =47.3%, WC = 3.9%; in 2004: BMI = 10.8%, WHR =59.4%, WC = 14.0%; in 2009: BMI = 26.6%, WHR =70.7%, WC = 39.7% | in 2009: BMI: male = 4.1%, Female = 4.6%; WHR: male = 31.3%, Female = 59.8%; WC: male = 1.9%, Female = 5.4%; in 2004: BMI: male = 9.6%, Female = 11.6%; WHR: male = 59.4%, Female = 39.3%; WC: male = 4.8%, Female = 20.2% ; in 2009: BMI: male = 25.1%, Female = 27.3%; WHR: male = 57.3%, Female = 77.6%; WC: male = 24.1%, Female = 48.2%; | not reported |
| Bhowmik, B., et al.,; 2013 [32] | Cross-sectional | Total: 2293 Chandra, Study period: March-Dec 2009 | Rural, age ≥ 20, | BMI (>25kg/m2); WC (men ≥ 90 cm and female ≥ 80 cm); WHR (men ≥ 0.90 and female ≥ 0.80); WHtR ≥0.50 | BMI = 24.4%, WHR =69.8%, WC = 37.9%, WHtR = 58.1% | BMI: male = 25.2%, Female = 26.8%; WHR: male = 58.6%, Female = 79.1%; WC: male = 24.4%, Female = 48.7%; WHtR: male = 53.5%, Female = 64.0% | not reported |
| Saquib N, et al.; 2013 [33] | Cross-sectional | Total: 402 Dhaka Study period: not mentioned | Urban, age ≥30 | BMI (>25kg/m2) | BMI = 63.1% | Male = 50.7% & Female = 76.7% | not reported |
| Karim A, et al., 2014[35] | Cross-sectional | Total: 1134Bangaon union,Savar Study period: August 2006-April 2009 | Rural, age =18-65 | BMI (>25kg/m2) | BMI = 20.1% | Not reported | not reported |

**References**

1. West KM, Kalbfleisch JM. Glucose tolerance, nutrition, and diabetes in Uruguay, Venezuela, Malaya, and East Pakistan. Diabetes. 1966 Jan;15(1):9-18. PubMed PMID: 5907153.
2. Mahtab H, Ibrahim M, Banik NG, Gulshan EJ, Haque F, Ali SM. Diabetes detection survey in a rural and a semiurban community in Bangladesh. Tohoku J Exp Med. 1983 Dec;141 Suppl:211-7. PubMed PMID: 6680489.
3. Sayeed MA, Khan AR, Banu A, Hussain MZ, Ali SM. Blood pressure and glycemic status in relation to body mass index in a rural population of Bangladesh. Bangladesh Med Res Counc Bull. 1994 Aug;20(2):27-35. PubMed PMID: 7748142.
4. Abu Sayeed M, Banu A, Khan AR, Hussain MZ. Prevalence of diabetes and hypertension in a rural population of Bangladesh. Diabetes Care. 1995 Apr;18(4):555-8. PubMed PMID: 7497870.
5. Sayeed MA, Hussain MZ, Banu A, Rumi MAK, Azad Khan AK. Prevalence of diabetes in a suburban population of Bangladesh. Diabetes Res Clin Pract. 1997 Jan;34(3):149-55. doi: http://dx.doi.org/10.1016/S0168-8227%2896%2901337-X. PubMed PMID: 9069566.
6. Sayeed MA, Ali L, Hussain MZ, Rumi MAK, Banu A, Khan AKA. Effect of socioeconomic risk factors on the difference in prevalence of diabetes between rural and urban populations in Bangladesh. Diabetes Care. 1997 April;20(4):551-5. PubMed PMID: 1997099947.
7. Zaman MM, Yoshiike N, Rouf MA, Syeed MH, Khan MR, Haque S, et al. Cardiovascular risk factors: distribution and prevalence in a rural population of Bangladesh. J Cardiovasc Risk. 2001 Apr;8(2):103-8. PubMed PMID: 11324369.
8. Sayeed MA, Mahtab H, Latif ZA, Khanam PA, Ahsan KA, Banu A, et al. Waist-to-height ratio is a better obesity index than body mass index and waist-to-hip ratio for predicting diabetes, hypertension and lipidemia. Bangladesh Med Res Counc Bull. 2003 Apr;29(1):1-10. PubMed PMID: 14674615.
9. Sayeed MA, Mahtab H, Akter Khanam P, Abdul Latif Z, Keramat Ali SM, Banu A, et al. Diabetes and impaired fasting glycemia in a rural population of Bangladesh. Diabetes Care. 2003 Apr;26(4):1034-9. PubMed PMID: 12663569.
10. Sayeed MA, Mahtab H, Khanam PA, Ahsan KA, Banu A, Rashid ANMB, et al. Diabetes and Impaired Fasting Glycemia in the Tribes of Khagrachari Hill Tracts of Bangladesh. Diabetes Care. 2004 May;27(5):1054-9. doi: http://dx.doi.org/10.2337/diacare.27.5.1054. PubMed PMID: 2004196768.
11. Sayeed MA, Mahtab H, Khanam PA, Begum R, Banu A, Azad Khan AK. Diabetes and hypertension in pregnancy in a rural community of Bangladesh: a population-based study. Diabet Med. 2005 Sep;22(9):1267-71. PubMed PMID: 16108860.
12. Rahman MM, Rahim MA, Nahar Q. Prevalence and risk factors of type 2 diabetes in an urbanizing rural community of Bangladesh. Bangladesh Med Res Counc Bull. 2007 Aug;33(2):48-54. PubMed PMID: 18481438.
13. Zaman MM, Ahmed J, Choudhury SR, Numan SM, Parvin K, Islam MS. Prevalence of ischemic heart disease in a rural population of Bangladesh. Indian Heart J. 2007 May-Jun;59(3):239-41. PubMed PMID: 19124932.
14. Sayeed MA, Mahtab H, Khanam PA, Latif ZA, Banu A, Khan AK. Prevalence of diabetes and impaired fasting glucose in urban population of Bangladesh. Bangladesh Med Res Counc Bull. 2007 Apr;33(1):1-12. Epub 2008/02/06. PubMed PMID: 18246729.
15. Rahim MA, Hussain A, Azad Khan AK, Sayeed MA, Keramat Ali SM, Vaaler S. Rising prevalence of type 2 diabetes in rural Bangladesh: a population based study. Diabetes Res Clin Pract. 2007 Aug;77(2):300-5. Epub 2006/12/26. doi: 10.1016/j.diabres.2006.11.010. PubMed PMID: 17187890.
16. Hussain A, Vaaler S, Sayeed MA, Mahtab H, Ali SM, Khan AK. Type 2 diabetes and impaired fasting blood glucose in rural Bangladesh: a population-based study. Eur J Public Health. 2007 Jun;17(3):291-6. doi: 10.1093/eurpub/ckl235. PubMed PMID: 17008328.
17. Hoang Van Minh M, Ng N, Juvekar S, Razzaque A, Ashraf A, Hadi A, et al. Peer Reviewed: Self-Reported Prevalence of Chronic Diseases and Their Relation to Selected Sociodemographic Variables: A Study in INDEPTH Asian Sites, 2005. Prev Chronic Dis. 2008 5(3):(http://www.cdc.gov/pcd/issues/2008/jul/07_0115.htm). PubMed PMID: http://www.cdc.gov/pcd/issues/2008/jul/07_0115.htm.
18. Sayeed S, Banu A, Khanam PA, Alauddin S, Makbul S, Begum T, et al. Prevalence of Metabolic Syndrom in Three Urban Communities of Dhaka City. Ibrahim Medical College Journal. 2008 2(2):44-8.
19. Bulletin ICDDRB. Type 2 diabetes and prediabetic conditions among adults aged 27-50 years in Matlab: a hidden public health burden. HSB. 2009 7(2).
20. Ahsan SA, Haque KS, Salman M, Bari AS, Nahar H, Ahmed MK, et al. Detection of ischaemic heart disease with risk factors in different categories of employees of University Grants Commission. University Heart Journal. 2009 5(1):20-3.
21. Rahim MA, Khan AKA, Ali SMK, Nahar Q, Shaheen A, Hussain A. Glucose tolerance in rural population of Bangladesh. Diabetes and Metabolic Syndrome: Clin Res Rev. 2009 Jan/March;3(1):24-8. doi: http://dx.doi.org/10.1016/j.dsx.2008.10.005. PubMed PMID: 2009142633.
22. Rahim MA, Azad Khan AK, Nahar Q, Ali SMK, Hussain A. Impaired fasting glucose and impaired glucose tolerance in rural population of Bangladesh. Bangladesh Med Res Counc Bull. 2010 Aug;36(2):47-51. PubMed PMID: 2011068100.
23. Parr JD, Lindeboom W, Khanam MA, Perez Koehlmoos TL. Diagnosis of chronic conditions with modifiable lifestyle risk factors in selected urban and rural areas of Bangladesh and sociodemographic variability therein. BMC Health Serv Res. 2011 11(1):309. doi: 10.1186/1472-6963-11-309. PubMed PMID: 22078128; PubMed Central PMCID: PMCPMC3239323.
24. Boffetta P, McLerran D, Chen Y, Inoue M, Sinha R, He J, et al. Body mass index and diabetes in Asia: a cross-sectional pooled analysis of 900,000 individuals in the Asia cohort consortium. PLoS One. 2011 6(6):e19930. doi: 10.1371/journal.pone.0019930. PubMed PMID: 21731609; PubMed Central PMCID: PMCPMC3120751.
25. Ahasan HAMN, Islam MZ, Alam MB, Miah MT, Zannatun N, Mohammed FR, et al. Prevalence and risk factors of type 2 diabetes mellitus among secretariat employees of Bangladesh. Journal of Medicine. 2011 12(2):125-30.
26. Akhter A, Fatema K, Afroz A, Bhowmik B, Ali L, Hussain A. Prevalence of diabetes mellitus and its associated risk indicators in a rural Bangladeshi population. Open Diab J. 2011 4(1):6-13. doi: http://dx.doi.org/10.2174/1876524601104010006. PubMed PMID: 2011553710.
27. Rahim MA, Vaaler S, Keramat Ali SM, Khan AK, Hussain A, Nahar Q. Prevalence of type 2 diabetes in urban slums of Dhaka, Bangladesh. Bangladesh Med Res Counc Bull. 2004 Aug;30(2):60-70. PubMed PMID: 15813484.
28. Das M, Hassan Z, Faruque O, Parial R, Khalequzzaman M, Ali L. Prevalence of abnormal glycemic and lipidemic status in an urban population of Bangladesh. J Bio-Sci. 2012 19:1-6.
29. Cravedi P, Sharma SK, Bravo RF, Islam N, Tchokhonelidze I, Ghimire M, et al. Preventing renal and cardiovascular risk by renal function assessment: Insights from a cross-sectional study in low-income countries and the USA. BMJ Open. 2012 2(5):e001357. doi: 10.1136/bmjopen-2012-001357. PubMed PMID: 23002161; PubMed Central PMCID: PMCPMC3467605.
30. Bhowmik B, Diep LM, Munir SB, Rahman M, Wright E, Mahmood S, et al. HbA(1c) as a diagnostic tool for diabetes and pre-diabetes: the Bangladesh experience. Diabetic Med. 2013 Mar;30(3):e70-7. doi: http://dx.doi.org/10.1111/dme.12088. PubMed PMID: 23199158.
31. Bhowmik B, Afsana F, Diep LM, Munir SB, Wright E, Mahmood S, et al. Increasing prevalence of type 2 diabetes in a rural Bangladeshi population: A population based study for 10 years (Diabetes Metab J 2013;37:46-53). Diab Metab J. 2013 April;37(2):153-4. doi: http://dx.doi.org/10.4093/dmj.2013.37.2.153. PubMed PMID: 2013312538.
32. Bhowmik B, Munir SB, Diep LM, Siddiquee T, Habib SH, Samad MA, et al. Anthropometric indicators of obesity for identifying cardiometabolic risk factors in a rural Bangladeshi population. J Diab Invest. 2013 July;4(4):361-8. doi: http://dx.doi.org/10.1111/jdi.12053. PubMed PMID: 24843680; PubMed Central PMCID: PMCPMC4020230.
33. Saquib N, Khanam MA, Saquib J, Anand S, Chertow GM, Barry M, et al. High prevalence of type 2 diabetes among the urban middle class in Bangladesh. BMC Public Health. 2013 13(1):1032.
34. Akter S, Rahman MM, Abe SK, Sultana P. Prevalence of diabetes and prediabetes and their risk factors among Bangladeshi adults: a nationwide survey. Bull World Health Organ. 2014 92(3):204-13. doi: http://dx.doi.org/10.2471/BLT.13.128371.
35. Karim A, Das D, Salahuddin M, Marjan G, Islam M, Shaha A, et al. Prevalence of Microalbuminuria and Overt Proteinuria in Hypertension and Their Relations with Renal Function in a Rural Population of Bangladesh. Bangladesh Journal of Medicine. 2014 24(2):59-64.
36. Malik A. Congenital and acquired heart diseases: (A survey of 7062 persons). Bangladesh Med Res Counc Bull. 1976 Dec;2(2):115-9. PubMed PMID: 1037368.
37. Ullah W. Hypertension in a mixed community. Bangladesh Med Res Counc Bull. 1976 Dec;2(2):95-9. PubMed PMID: 1037377.
38. Islam N. Hypertension in secretariat population of Bangladesh. Bangladesh Med Res Counc Bull. 1979 Jun;5(1):19-24. PubMed PMID: 550817.
39. Islam N, Khan M, Latif ZA. Hypertension in the rural population of Bangladesh--a preliminary survey. Bangladesh Med Res Counc Bull. 1983 Jun;9(1):11-4. PubMed PMID: 6670965.
40. Abu Sayeed M, Banu A, Malek MA, Azad Khan AK. Blood pressure and coronary heart disease in NIDDM subjects at diagnosis: Prevalence and risks in a Bangladeshi population. Diab Res Clin Prac. 1998 Feb;39(2):147-55. doi: http://dx.doi.org/10.1016/S0168-8227%2898%2900004-7. PubMed PMID: 1998167334.
41. Hypertension Study G. Prevalence, awareness, treatment and control of hypertension among the elderly in Bangladesh and India: a multicentre study. Bull of the World Health Organization. 2001 79(6):490-500. PubMed PMID: 11436469; PubMed Central PMCID: PMCPMC2566443.
42. Sayeed MA, Banu A, Haq JA, Khanam PA, Mahtab H, Azad Khan AK. Prevalence of hypertension in Bangladesh: Effect of socioeconomic risk factor on difference between rural and urban community. Bangladesh Med Res Counc Bull. 2002 April;28(1):7-18. PubMed PMID: 2003047184.
43. Zaman MM, Choudhury SR, Ahmed J, Numan SM, Islam MS, Yoshiike N. Non-biochemical risk factors for cardiovascular disease in general clinic-based rural population of Bangladesh. J Epidemiol. 2004 Mar;14(2):63-8. PubMed PMID: 15162980.
44. Chen Y, Factor-Litvak P, Howe GR, Parvez F, Ahsan H. Nutritional influence on risk of high blood pressure in Bangladesh: a population-based cross-sectional study. Am J Clin Nutr. 2006 84(5):1224-32. PubMed PMID: 17093178.
45. Ahmed S, Shirin S, Mohsena M, Parvin N, Sultana N, Sayed S, et al. Geriatric health problems in a rural community of Bangladesh. Ibrahim Medical College Journal. 2007 1(2):17-20.
46. Van Minh H, Soonthornthada K, Ng N, Juvekar S, Razzaque A, Ashraf A, et al. Blood pressure in adult rural INDEPTH population in Asia. Glob Health Action. 2009 2. doi: 10.3402/gha.v2i0.2010. PubMed PMID: 20027254; PubMed Central PMCID: PMCPMC2785103.
47. Moni MA, Rahman MA, Haque MA, Islam MS, Ahmed K. Blood pressure in relation to selected anthropometric measurements in senior citizens. Mymensingh Med J. 2010 Apr;19(2):254-8. PubMed PMID: 20395922.
48. Das S, Dutta P. Chronic kidney disease prevalence among health care providers in Bangladesh. Mymensingh Med J: MMJ. 2010 19(3):415-21.
49. Islam MR, Khan I, Attia J, Hassan SMN, McEvoy M, D'Este C, et al. Association between hypertension and chronic arsenic exposure in drinking water: A cross-sectional study in Bangladesh. Int J Environ Res Public Health. 2012 Dec;9(12):4522-36. doi: http://dx.doi.org/10.3390/ijerph9124522. PubMed PMID: 2012755546.
50. Ahmed A, Rahman M, Hasan R, Shima SA, Faruquee M, Islam T, et al. Hypertension and associated risk factors in some selected rural areas of Bangladesh. Intern J Rese Medical Sci. 2014 2(3):925-31.
51. Zaman MM, Choudhury SR, Ahmed J, Yoshiike N, Numan SM, Islam MS, et al. Plasma lipids in a rural population of Bangladesh. Eur J Cardiovasc Prev Rehabil. 2006 June;13(3):444-8. doi: http://dx.doi.org/10.1097/00149831-200606000-00022. PubMed PMID: 2006425431.
52. Shekhar HU, Shahjalal HM, Ahmed R, Uddin M, Kaniz K-E-J. Prevalence of Dyslipidemic Phenotypes Including Hyper-apoB and Evaluation of Cardiovascular Disease Risk in Normocholesterolemic Type 2 Diabetic Patients. Pak J Biol Sci. 2006 9:1536-41.
53. Alam MB, Ahasan HN, Islam MZ, Islam MN, Mohammed FR, Nur Z, et al. Pattern of lipid profile and obesity among secretariat employees of Bangladesh. Journal of Medicine. 2009 10(3):3-6.
54. Afsana F, Latif ZA, Khan SJ, Talukder SK. Metabolic syndrome and cardiovascular risk in diabetic subjects. CVD Prevention and Control. 2010 June;5(2):59-62. doi: http://dx.doi.org/10.1016/j.cvdpc.2010.05.001. PubMed PMID: 2010503376.
55. Das SK, Golam Faruque AS, Chowdhury AK, Chisti MJ, Hossain MA, Salam MA, et al. Lipoprotein status among urban populations in Bangladesh. Atherosclerosis. 2012 223(2):454-7.
56. Islam N, Rahman MZ, Choudhury S, Afrin L, Rahman S, Aftabuddin M. Prevalence of Dyslipidemia and Associated Factors among the Sub-Urban Bangladeshi Population. University Heart Journal. 2012 8(1):15-9.
57. Cohen N, Measham AR, Akbar J. Smoking and respiratory disease symptoms in rural Bangladesh. Public Health. 1983 Nov;97(6):338-46.
58. Islam N, Islam MN, Khanam K. Smoking habit among Bangladesh Secretariat staff. Bangladesh Med Res Counc Bull. 1990 Dec;16(2):62-9.
59. Ahsan H, Underwood P, Atkinson D. Smoking among male teenagers in Dhaka, Bangladesh. Prev Med. 1998 27(1):70-6. doi: http://dx.doi.org/10.1006/pmed.1997.0239.
60. Khan MMH, Aklimunnessa K, Kabir MA, Kabir M, Mori M. Tobacco consumption and its association with illicit drug use among men in Bangladesh. Addiction. 2006 101(8):1178-86. doi: http://dx.doi.org/10.1111/j.1360-0443.2006.01514.x.
61. Choudhury K, Hanifi SM, Mahmood SS, Bhuiya A. Sociodemographic characteristics of tobacco consumers in a rural area of Bangladesh. J Health Pop Nutr. 2007 Dec;25(4):456-64. PubMed PMID: 18402189; PubMed Central PMCID: PMCPMC2754020.
62. Rahman M, Awal ASMN, Fukui T, Sakamoto J. Prevalence of cigarette and bidi smoking among rickshaw pullers in Dhaka city. Prev Med. 2007 Mar;44(3):218-22. Epub 2006/12/19. doi: 10.1016/j.ypmed.2006.11.001. PubMed PMID: 17173963.
63. Khan MMH, Khan A, Kraemer A, Mori M. Prevalence and correlates of smoking among urban adult men in Bangladesh: Slum versus non-slum comparison. BMC Public Health. 2009 9(149). doi: http://dx.doi.org/10.1186/1471-2458-9-149. PubMed PMID: 2009376159.
64. Ashraf A, Quaiyum MA, Ng N, Van Minh H, Razzaque A, Masud Ahmed S, et al. Self-reported use of tobacco products in nine rural INDEPTH Health and Demographic Surveillance Systems in Asia. Glob Health Action. 2009 Sep;2. Epub 2009/12/23. doi: 10.3402/gha.v2i0.1997. PubMed PMID: 20027256; PubMed Central PMCID: PMCPMC2785137.
65. Mostafa MG, McDonald JC, Cherry N. Lung cancer and exposure to arsenic in rural Bangladesh. Occup Environ Med. 2008 Nov;65(11):765-8. doi: 10.1136/oem.2007.037895. PubMed PMID: 18417558.
66. Flora MS, Mascie-Taylor CGN, Rahman M. Gender and locality differences in tobacco prevalence among adult Bangladeshis. Tob Control. 2009 18(6):445-50.
67. Siddiqui MNA, Sultana S, Sharif T, Ekram ARMS. Smoking habits of medical students in a private medical college of Bangladesh. Bangladesh Journal Medical Sciences. 2011 10(4):280-3. PubMed PMID: 2012068238.
68. Kamal SM, Islam MA, Rahman MA. Sociopsychological correlates of smoking among male university students in Bangladesh. Asia Pac J Public Health. 2011 Jul;23(4):555-67. doi: http://dx.doi.org/10.1177/1010539509350495. PubMed PMID: 20460274.
69. Pesola GR, Parvez F, Chen Y, Ahmed A, Hasan R, Ahsan H. Arsenic exposure from drinking water and dyspnoea risk in Araihazar, Bangladesh: a population-based study. Eur Respir J. 2012 May;39(5):1076-83. doi: 10.1183/09031936.00042611. PubMed PMID: 22088973; PubMed Central PMCID: PMCPMC3955754.
70. Razzak A. A questionnaire survey on infectious disease among hospital patients in Kushtia and Jhenaidah, Bangladesh. Int J Genet Mol Biol. 2011 Oct;3(9):120-34.
71. Hanifi SM, Mahmood SS, Bhuiya A. Smoking has declined but not for all: findings from a study in a rural area of Bangladesh. Asia Pac J Public Health. 2011 Sep;23(5):662-71. doi: http://dx.doi.org/10.1177/1010539509351051. PubMed PMID: 20498124.
72. Razzaque A, Nahar L, Mustafa AHMG, Ahsan KZ, Islam MS, Yunus M. Sociodemographic differentials of selected noncommunicable diseases risk factors among adults in Matlab, Bangladesh: findings from a WHO STEPS survey. Asia Pac J Public Health. 2011 23(2):183-91.
73. Sinha DN, Gupta PC, Ray C, Singh PK. Prevalence of smokeless tobacco use among adults in WHO South-East Asia. Indian J Cancer. 2012 Oct-Dec;49(4):342-6. doi: http://dx.doi.org/10.4103/0019-509X.107726. PubMed PMID: 2013226801.
74. Palipudi KM, Sinha DN, Choudhury S, Zaman MM, Asma S, Andes L, et al. Predictors of tobacco smoking and smokeless tobacco use among adults in Bangladesh. Indian J Cancer. 2012 Oct-Dec;49(4):387-92. doi: http://dx.doi.org/10.4103/0019-509X.107745. PubMed PMID: 2013226808.
75. Kishore J, Jena PK, Bandyopadhyay C, Swain M, Das S, Banerjee I. Hardcore smoking in three South-East asian countries: results from the global adult tobacco survey. Asian Pac J Cancer Prev. 2013 14(2):625-30. PubMed PMID: 23621209.
76. Kabir MA, Goh KL, Kamal SM, Khan MM. Tobacco smoking and its association with illicit drug use among young men aged 15-24 years living in urban slums of Bangladesh. PLoS ONE [Electronic Resource]. 2013 8(7):e68728. doi: http://dx.doi.org/10.1371/journal.pone.0068728. PubMed PMID: 23935885; PubMed Central PMCID: PMCPMC3728353.
77. Kabir MA, Goh KL, Khan MM. Tobacco consumption and illegal drug use among Bangladeshi males: association and determinants. Am J of Mens Health. 2013 Mar;7(2):128-37. doi: http://dx.doi.org/10.1177/1557988312462737. PubMed PMID: 23065136.
78. Bartlett E, Parr J, Lindeboom W, Khanam MA, Koehlmoos TP. Sources and prevalence of self-reported asthma diagnoses in adults in urban and rural settings of Bangladesh. Glob Public Health. 2013 8(1):79-89. doi: 10.1080/17441692.2012.758761. PubMed PMID: 23305210.
79. Sreeramareddy CT, Pradhan PMS, Mir IA, Shwe S. Smoking and smokeless tobacco use in nine South and Southeast Asian countries: prevalence estimates and social determinants from Demographic and Health Surveys. Popul Health Metr. 2014 Aug 28;12:22. Epub 2014/09/04. doi: 10.1186/s12963-014-0022-0. PubMed PMID: 25183954; PubMed Central PMCID: PMCPMC4151025.
80. Sayeed MA, Mahtab H, Sayeed S, Begum T, Khanam PA, Banu A. Prevalence and risk factors of coronary heart disease in a rural population of Bangladesh. Ibrahim Medical College Journal. 2010 4(2):37-43.
81. Mohammad QD, Habib M, Hoque A, Alam B, Haque B, Hossain S, et al. Prevalence of stroke above forty years. Mymensingh Med J. 2011 Oct;20(4):640-4. PubMed PMID: 22081183.
82. Zaman MM, Yoshiike N. Prevalence of overweight defined by body mass index in a rural adult population of Bangladesh. J Health Pop Nutr. 2003 Jun;21(2):162-3. PubMed PMID: 13677444.
